# Terpenoids: Emerging Natural Modulators for Reversing ABC Transporter-Mediated Multidrug Resistance in Cancer Chemotherapy

**DOI:** 10.3390/ph19010146

**Published:** 2026-01-14

**Authors:** Lanfei Ma, Dina Mahemuti, Yuanhong Lan, Jianxiong Xu, Wenfang Li, Zhengding Su, Jinyao Li, Aytursun Abuduwaili, Ayitila Maimaitijiang

**Affiliations:** 1 School of Pharmaceutical Science, Institute of Materia Medica, College of Life Science and Technology, Xinjiang University, Urumqi 830017, China; 107552403479@stu.xju.edu.cn (L.M.); dina333@stu.xju.edu.cn (D.M.); liwenfang@xju.edu.cn (W.L.); james_su@xju.edu.cn (Z.S.); ljyxju@xju.edu.cn (J.L.); 2 College of Chemistry and Chemical Engineering, Xinjiang Agricultural University, Urumqi 830052, China

**Keywords:** terpenoids, tumor multidrug resistance, reversal, pharmacological activity

## Abstract

Multidrug resistance (MDR) is a central cause of chemotherapy failure and tumor recurrence and metastasis, and its mechanism involves enhanced drug efflux, target mutation, upregulation of DNA repair and remodeling of the tumor microenvironment. ABC transporter protein (P-gp, MRP, and BCRP)-mediated efflux of drugs is the most intensively researched aspect of the study, but the first three generations of small-molecule reversal agents were stopped in the clinic because of toxicity or pharmacokinetic defects. Natural products are considered as the fourth generation of MDR reversal agents due to their structural diversity, multi-targeting and low toxicity. In this paper, we systematically summarize the inhibitory activities of monoterpenes, sesquiterpenes, diterpenes and triterpenes against ABC transporter proteins in in vitro and in vivo models and focus on the new mechanism of reversing drug resistance by blocking efflux pumps, modulating signaling pathways such as PI3K-AKT, Nrf2, NF-*κ*B and remodeling the tumor microenvironment. For example, Terpenoids possess irreplaceable core advantages over traditional multidrug resistance (MDR) reversers: Compared with the first three generations of synthetic reversers, natural/semisynthetic terpenoids integrate low toxicity (mostly derived from edible medicinal plants, half-maximal inhibitory concentration IC_50_ > 50 μM), high target specificity (e.g., oleanolic acid specifically inhibits the ATP-binding cassette (ABC) transporter subtype ABCC1 without cross-reactivity with ABCB1), and multi-mechanistic synergistic effects (e.g., *β*-caryophyllene simultaneously mediates the dual effects of “ABCB1 efflux inhibition + apoptotic pathway activation”). These unique characteristics enable terpenoids to effectively circumvent key limitations of traditional synthetic reversers, such as high toxicity and severe drug–drug interactions. Among them, lupane-type derivative BBA and euphane-type sooneuphanone D (triterpenoids), as well as dihydro-*β*-agarofuran-type compounds and sesquiterpene lactone Conferone (sesquiterpenoids), have emerged as the core lead compounds with the greatest translational potential in current MDR reverser research, attributed to their potent in vitro and in vivo MDR reversal activity, low toxicity, and excellent druggable modifiability. At the same time, we point out bottlenecks, such as low bioavailability, insufficient in vivo evidence, and unclear structure–activity relationship and put forward a proposal to address these bottlenecks. At the same time, the bottlenecks of low bioavailability, insufficient vivo evidence and unclear structure–activity relationship have been pointed out, and future research directions such as nano-delivery, structural optimization and combination strategies have been proposed to provide theoretical foundations and potential practical pathways for the clinical translation research of terpenoid compounds, whose clinical application still requires further in vivo validation and translational research support.

## 1. Introduction

Cancer is of the most common causes of death and is a major public health safety issue facing the world [1]. Scientists have analyzed data from the International Agency for Research on Cancer (IARC) and predicted that by 2050, the number of new cancer cases is expected to reach 35.3 million globally; at the same time, the number of cancer deaths is expected to reach 18.5 million [2,3]. In the field of cancer treatment, chemotherapy, as the most core treatment tool, is widely used in various types of cancer [4]. However, its efficacy is frequently compromised by the emergence and progression of multidrug resistance. Drug resistance not only exacerbates tumor invasiveness and promotes the metastatic process, but also markedly diminishes the effectiveness of chemotherapy regimens. Tumor multidrug resistance denotes the phenomenon whereby chemotherapeutic agents of varying structures and functions exhibit reduced efficacy or become ineffective against tumor cells [5,6]. At present, with the in-depth study of tumor resistance mechanisms by scientists, four generations of multidrug resistance reversal agents have been developed. However, three generations of MDR inhibitors have significant limitations in terms of toxicity, dosage, drug metabolism, and clinical efficacy; none of them have been successfully applied in clinical treatment [7]. With the deepening of research, scientists have found that natural products are regarded as the fourth generation of MDR reversal agents, and they show great potential in reversing multidrug resistance in tumors due to their novel structure, multi-target action and low toxicity. These reversal agents mainly include compounds such as flavonoids, alkaloids, terpenoids, polyphenols, and coumarins, which not only have low toxicity but also exhibit significant MDR-modulating activities [6,8].

In recent years, terpenoids have gained extensive attention as promising multidrug resistance (MDR) reversers due to their unique chemical structures. This review systematically summarizes their activity profiles, mechanisms of action, combination therapy strategies, and delivery systems while discussing current challenges and future directions in this field. Terpenoids hold two pivotal advantages: highly diverse chemical scaffolds enabling multi-target synergistic effects and excellent structural plasticity. These features not only overcome the limitations of single-target inhibition but also play a role as a critical foundation for rationally designing precision-regulated MDR reversers with high selectivity and favorable pharmacokinetic (PK) properties, thereby facilitating the transition from broad-spectrum intervention to target-specific modulation.

## 2. Mechanisms of MDR

**Figure 1:**
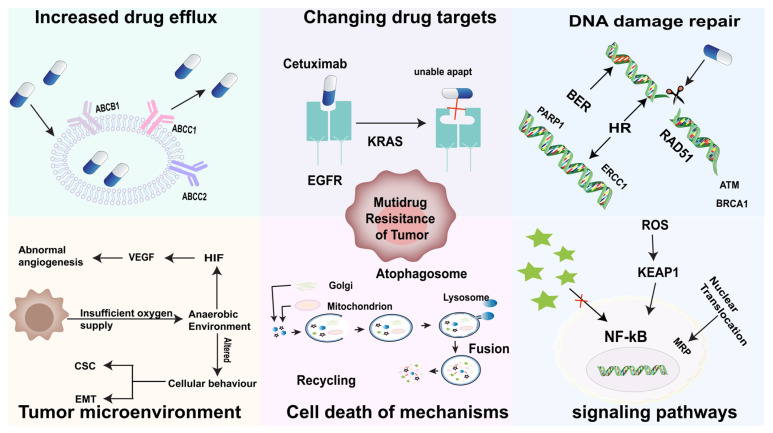
Mechanisms of Multidrug Resistance in Cancer. The mechanisms of multidrug resistance in cancer include increased drug efflux, altered drug targets, enhanced DNA damage repair, cell death-mediated MDR, MDR-associated factors or signaling pathways, and tumor microenvironment effect. ABCB1, ATP-Binding Cassette Subfamily B Member 1; ABCC1, ATP-Binding Cassette Subfamily C Member 1; ABCC2, ATP-Binding Cassette Subfamily C Member 2. EGFR, Epidermal Growth Factor Receptor, regulates cell proliferation, differentiation, migration, and apoptosis. KRAS, Kirsten Rat Sarcoma Viral Oncogene Homolog, is a key downstream molecule in the EGFR pathway, switching between the GTP-bound active state and the GDP-bound inactive state, thereby regulating cell proliferation and survival. BER, Base Excision Repair; HR, Homologous Recombination. High-precision DNA double-strand break (DSB) repair pathways utilize sister chromatids as templates for damage repair, thereby safeguarding the integrity of genetic material. PARP1, polyadenine diphosphate ribose polymerase 1. In tumors with defective HR pathways, inhibiting PARP1 leads to unrepaired DNA damage and subsequent cell death, known as synthetic lethality. This represents a key therapeutic target in cancers such as ovarian and breast cancer. ERCC1, excision repair cross-complementing group 1, is the core molecule of the nucleotide excision repair (NER) pathway, involved in repairing intranuclear acid cross-linking damage caused by ultraviolet radiation and chemotherapeutic agents such as cisplatin. RAD51, the DNA repair protein RAD51 homolog, serves as the central recombinase in the homologous recombination repair pathway. Under the regulation of molecules such as ATM, it mediates the insertion of single-stranded DNA into the homologous template strand, thereby accomplishing the high-precision repair of double-strand breaks. ATM, Ataxia—Telangiectasia Mutated, is a core regulatory molecule in the DNA damage response, and it detects double-strand breaks in DNA and activates downstream kinases such as CHK1/CHK2, thereby initiating cell cycle checkpoints, DNA repair, or apoptosis programs. BRCA1, Breast Cancer gene 1, is an oncogene that participates in homologous recombination repair, cell cycle regulation, and transcriptional control. ROS, Reactive Oxygen Species, are oxygen-containing active molecules produced by aerobic metabolism within cells (such as superoxide anion and hydrogen peroxide). Low levels of ROS participate in cellular signaling, whilst high levels of ROS can cause oxidative damage to DNA, proteins, and lipids. KEAP1, Kelch-like ECH-associated protein 1, is a core regulatory molecule in the oxidative stress response pathway, which can bind to Nrf2 and mediate its ubiquitination and degradation. NF-KB, Nuclear Factor Kappa-Light-Chain-Enhancer of Activated B cells, participates in regulating immune inflammation, cell proliferation, apoptosis, and processes such as invasion and metastasis. MRP, Multidrug Resistance-Associated Protein, excretes chemotherapeutic agents, natural products, and other substances from within the cell to the extracellular space, thereby lowering extracellular drug concentrations and leading to multidrug resistance in tumors. HIF, Hypoxia-Inducible Factor, correlates positively with tumor malignancy and therapeutic resistance when showing elevated expression. VEGF, Vasc Vascular Endothelial Growth Factor, is a cytokine that acts explicitly upon vascular endothelial cells and promotes endothelial cell proliferation, migration, and angiogenesis, thereby supplying nutrients and oxygen to tumor growth. CSC, Cancer Stem Cell, can tolerate chemotherapy and radiotherapy while mediating tumor recurrence, metastasis, and multidrug resistance. EMT, Epithelial-to-Mesenchymal Transition, can enhance the invasive and migratory capabilities of tumor stem cell-like properties and multidrug resistance. Image created using Adobe Illustrator 2024 software.

The mechanisms of MDR during chemotherapy are multidirectional and include increased drug efflux, altered drug targeting, increased DNA damage repair, the tumor microenvironment, MDR-associated factors or signaling pathways, cell death, and regulation. The mechanisms of MDR development are extremely complex and may not be singular but rather multiple mechanisms that intricately regulate the development of tumor MDR, and we specifically discuss the mechanisms that induce tumor MDR development below (Figure 1).

### 2.1. Increased Drug Efflux

Tumor cells actively pump chemotherapeutic drugs out of the cell, thereby reducing the amount and effect of the drug within the cell [9]. ABC transporter proteins are the main drivers and the main cause of MDR. ABC transporter proteins consist of two distinct conserved structural domains, including nucleotide-binding structural domains (NBDs) and transmembrane structural domains (TMDs). The (NBDs) are in the cytoplasm and are responsible for the transport of substrates via membrane energy transfer, and the (TMDs) consist of six transmembrane alpha-helices that confer substrate specificity [10,11,12]. Proteins of the ABC transporter superfamily excrete drugs out of the cell and reduce the body’s sensitivity to chemotherapeutic drugs, thus allowing tumor cells to develop multidrug resistance [13,14]. These proteins cross the cell membrane by extruding a wide range of hydrophobic compounds and metabolites, which leads to a decrease in intracellular drug concentration [15,16]. Research has shown that as patients use chemotherapy drugs for an extended period, the expression of ABC transporters also increases, leading to an increase in tumor cell resistance [17].

In the process of MDR involving ABC transporters, it usually relies on the energy generated by ATP to maintain the continuous operation of the drug efflux pump, which is influenced by the activity of ABC transporters. There are seven ABC transporter superfamilies, the most studied ones mainly include P-gp protein (ABCB1) and ATP binding box subfamily C member/multidrug resistance related protein 1 (MRP1), multidrug resistance protein 2 (MRP2) protein, and breast cancer resistance protein (BCRP), which are involved in the multidrug resistance of different tumors [18].

### 2.2. Changing Drug Targets

Changes in expression levels or mutations in the targets of chemotherapeutic agents may significantly affect drug sensitivity [19]. Tumor resistance severely limits the clinical efficacy of kinase inhibitors as targeted cancer therapies. For instance, vemurafenib, erlotinib and crizotinib have demonstrated favorable outcomes in BRAF-mutated melanoma, EGFR-mutated or ALK-rearranged lung adenocarcinoma, respectively, with most patients initially exhibiting tumor shrinkage or stabilization. However, varying degrees of resistance frequently emerge within 6–12 months of treatment, with this tolerance to targeted therapy ultimately hindering sustained tumor control. Resistance typically originates from a minority of pre-existing, intrinsically resistant or rapidly adapting cellular subpopulations. Elucidating how this cohort survives and evolves during tumor progression represents a core scientific challenge in unraveling the mechanisms of resistance development [19,20,21]. Studies have reported the resistance of lung adenocarcinoma to Critinib, which is caused by an acquired mutation in codon 2032 of the kinase domain of reactive oxygen species (ROS) oncogene 1 (ROS1), which has a substitution from glycine to arginine. Similarly, secondary EGFR mutations in the external structural domain S492R lead to cetuximab resistance by preventing EGFR antibodies from binding to their target sites in colon cancer [22,23].

It has been found that the chemotherapeutic effect of 5-fluorouracil (5-FU) works through its metabolite FdUMP (floxuridine monophosphate), which is highly effective in inhibiting thymidine synthase (TS), thereby killing tumor cells. Numerous preclinical studies have demonstrated that TS is a key determinant of 5-FU sensitivity. Furthermore, in vitro studies have shown that a dramatic increase in TS expression induces resistance to TS inhibitors such as TDX, 5-FU and multi-target antifolate [24,25,26]. The mechanism of the chemotherapeutic agent itself or different drug target modifying factors that occur during the adaptive process of the organism is one of the most important causes of MDR in tumors.

### 2.3. Increased DNA Damage Repair

Numerous endogenous and exogenous DNA-damaging agents (e.g., chemotherapeutics, ultraviolet radiation, ionizing radiation [IR]) induce diverse DNA lesions, including base mismatches, single-strand breaks (SSBs), double-strand breaks (DSBs), base/glycan chemical modifications, and intra-/inter-strand cross-links. Unrepaired DNA damage can cause genomic instability and mutation accumulation. To counteract these risks, cells have evolved an elaborate repair network that executes DNA Damage Repair (DDR) responses via multiple pathways [27]. Due to homologous recombination DNA repair damage, tumor cells rely on alternative repair mechanisms and are subject to polymerase (PARP) inhibitors that specifically kill homologous recombination-deficient cancer cells and have emerged as candidates for targeted cancer therapy [28]. Chemotherapeutic agents that target replicating DNA can trigger the ATR-CHK1 signaling pathway [29,30,31]. PU-1, a sesquiterpenoid from the Asteraceae family, inhibits the proliferation of drug-resistant cancer cells by causing DNA damage, blocking cell cycle progression in the G2/M phase, and triggering apoptosis [32]. PARP family proteins (notably PARP1), core mediators of single-strand DNA damage repair, have inhibitors (e.g., olaparib) that selectively eliminate tumor cells with defective DNA damage repair via “synthetic lethality” [33]. Terpenoids (e.g., euphorbians, taxanes) not only directly induce extensive double-strand DNA damage in tumor cells but also impair their DNA repair capacity by downregulating ATR-CHK1 pathway-related proteins (CHK1, RAD51) [34,35]. The combination of these two agents creates a synergistic effect whereby PARP inhibitors block single-strand repair while terpenoid compounds exacerbate double-strand damage. This disrupts the DNA damage repair network of tumor cells at multiple points, thereby reversing their resistance to chemotherapeutic agents such as cisplatin and doxorubicin [35,36]. Preliminary research indicates that jatrophane-type diterpenoids isolated from *Euphorbiaceae* plants, when combined with olaparib, can increase the apoptosis rate of cisplatin-resistant non-small cell lung cancer A549/DDP cells to 2.3 times that of the monotherapy group, while significantly reducing the drug resistance of their in vivo transplanted tumors [37]. This finding provides crucial preclinical theoretical and experimental support for the reversal of tumor multidrug resistance through the combination of natural terpenoid compounds with PARP inhibitors.

### 2.4. Tumor Microenvironment (TME)

The tumor microenvironment (TME) is an intricate cellular and tissue network comprising fibroblasts, adipocytes, stromal cells, immune cells, endothelial cells, and their secreted extracellular matrix proteins, and it is closely associated with tumor growth and progression. Exosomes, a key component of the TME, are nanoscale vesicles that deliver proteins, lipids, and nucleic acids to target cells [38,39,40]. Their roles in facilitating tumor progression are multifaceted: they not only protect tumor cells by delivering proteins that enhance DNA repair capacity but also promote the expression of drug efflux pumps (e.g., P-glycoprotein, P-gp), thereby reducing intracellular chemotherapeutic drug accumulation and enabling tumor cell survival under chemotherapy [41,42,43]. In the hypoxic tumor microenvironment (TME), chemotherapy resistance is enhanced, drug-induced cellular damage is reduced, and cancer cell growth and metastasis are promoted. Several anticancer drugs (e.g., cisplatin [DDP], etoposide [VP-16], gemcitabine) require oxygen to exert maximal anticancer effects. Hypoxia-inducible factor (HIF) proteins are key mediators of chemotherapy resistance: under hypoxia, HIF-1*α* promotes P-glycoprotein (P-gp) translation and upregulates the expression of multidrug resistance (MDR)-associated proteins (MRP1, BCRP, LRP) [44,45].

Terpenoids can specifically target and reverse the aforementioned resistance mechanisms, primarily by inducing M2-to-M1 reprogramming (e.g., betulinic acid inhibits the mTOR pathway) [46]. Intervening in metabolic pathways (such as inhibiting OGT to reduce CTSB secretion) and suppressing exosome secretion (such as oleanolic acid downregulating Rab27a) disrupts the TME-promoted drug resistance network through multiple mechanisms [47,48,49]. These effects offer novel strategies for reversing multidrug resistance, but their structure–activity relationships and clinical translation require further validation.

### 2.5. MDR-Related Factors or Signaling Pathways

Tumor drug resistance is a complex process involving multiple genes and signaling pathways and is closely related to decreased drug uptake and increased efflux. For example, tumor cells may increase the expression of drug efflux pumps, such as P-glycoprotein (P-gp), through activation of certain signaling pathways, leading to reduced accumulation of chemotherapeutic drugs in the cell, and thus drug resistance [19]. The PI3K-Akt pathway plays a central role in cell growth, maturation and death. Its abnormal activation is directly associated with chemotherapy resistance in multiple tumors. The combined use of inhibitors targeting this pathway can effectively enhance chemotherapy efficacy and reduce the required drug concentrations [50,51,52]. Moreover, the transcription factor Nrf2 plays a pivotal role in tumor cell drug resistance and proliferation by regulating the expression of antioxidant enzymes and cell protection-related genes. Meanwhile, NF-κB, as a key transcription complex, modulates crucial biological processes including DNA transcription, cytokine production, and cell survival. Its activation is closely associated with apoptosis inhibition, tumor proliferation promotion, and the development of drug resistance [53,54]. Among the multiple mechanisms, activation of the “efflux pump” with high expression of ABC transporter proteins may be the final step leading to MDR, and changes in the pre-transcriptional and post-transcriptional levels of multiple transcription factors upstream of the MDR, which extends to the complex regulation of the signaling pathways, may be the key reason for the emergence of intrinsic or acquired drug resistance.

**Figure 2:**
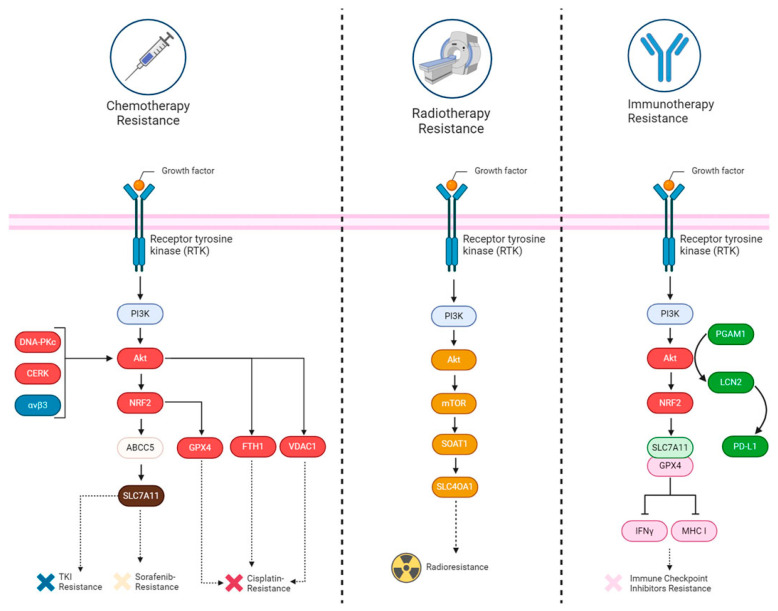
Signal pathways reversing tumor multidrug resistance. A summary of the mechanisms involved in acquiring therapy resistance (chemotherapy, immunotherapy, and radiotherapy) through the suppression of ferroptosis by the PI3K/Akt signaling pathway in cancers [55]. Reprinted with permission from Su et al., Frontiers in Cell and Developmental Biology, 2024, Frontiers Media S.A. © 2024, Frontiers Media S.A.

The high incidence and mortality rates of cancer necessitate novel therapeutic strategies. As a core signaling axis frequently abnormally activated in cancer, disruption of the PI3K/Akt pathway—such as through PIK3CA mutations or PTEN loss—constitutes a key driver of tumorigenesis, progression, and therapeutic resistance. This pathway influences cellular proliferation, apoptosis, and metabolism by regulating downstream signals such as Nrf2 and NF-κ*B*, alongside targets including mTOR and GSK3*β*. It also exhibits close interactions with novel cell death mechanisms such as ferroptosis. As illustrated in Figure 2, the connections between the three pathways are explained in detail [55,56].

### 2.6. Cell Death and Regulatory Mechanisms

Evidence from numerous studies suggests that MDR often follows the autophagic process. Enhanced autophagic activity observed in those patients with a poorer prognosis implies that autophagy may contribute to the formation of MDR [57,58,59]. Sun et al. demonstrated that Epirubicin induces autophagy in both Epirubicin-sensitive and resistant MCF-7 breast cancer cells. In resistant MCF-7/ER cells, autophagy activation enables cellular evasion of Epirubicin-induced apoptosis, functioning as a pro-survival mechanism that impairs apoptotic pathways. Further investigations on two ABCB1-overexpressing, Epirubicin-resistant cell lines (MCF-7/ER, SK-BR-3/ER)—which are cross-resistant to paclitaxel and vincristine—revealed that this chemotherapeutics also trigger autophagy, thereby protecting resistant cells from apoptotic death. Although ABCB1 expression is not correlated with autophagy, the role of autophagy in facilitating multidrug resistance (MDR) remains indispensable [60,61]. Researchers observed that the therapeutic effect of cisplatin on bladder cancer was enhanced after treating cisplatin-resistant bladder cancer cells with autophagy inhibitors [62,63].

## 3. Tumor MDR Reversal Agents

Above, we have focused on the mechanisms of MDR tumor occurrence. According to reports, three generations of reversal agents can overcome multidrug resistance in tumors. However, due to toxicity or pharmacokinetic issues, the first three generations of inhibitors have not been clinically advanced; the development of the next generation of reversal agents will require the involvement of more people. So far, no reversal agent has been successfully approved by the FDA and widely used to treat multidrug resistance in tumors. We have summarized generations of multidrug resistance reversal agents and their toxicities, side effects and structures, as shown in Table 1.

To break through multidrug resistance during chemotherapy, scientists have so far investigated four classes of reversal agents. The first category includes verapamil, cyclosporine A, rifampicin, quinidine and tamoxifen; however, it proved ineffective due to a narrow therapeutic window, significant toxicity at higher doses, and insufficient P-gp inhibitory activity, leading to severe adverse reactions such as cardiovascular suppression and neurotoxicity [64]. The second generation of inhibitors, including R-verapamil and Valspodar, are modifications of the first generation and inhibit other ABC transporter proteins; although the affinity of the second-generation inhibitors has been improved, they have been found to have an inhibitory effect on drug metabolizing enzymes, resulting in abnormally elevated plasma concentrations of chemotherapy drugs beyond safe limits, triggering life-threatening cumulative toxicity [66,67]. The third generation of inhibitors, including zosuquidar, laniquidar (R101933) and tariquidar (XR9576), is a group of highly selective inhibitors produced by scientists on the basis of quantitative conformational relationships and histology, but unsatisfactorily, it has the same defect of high toxicity as the first and second generations of inhibitors, and it also resulted in clinical failure. Although third-generation agents achieve high selectivity through quantitative structure–activity relationships and histological design, they remain constrained by high toxicity (as exemplified by tariquidar, whose toxicity led to the discontinuation of its Phase III clinical trial for lung cancer), significantly limiting their clinical utility [70,71]. In summary, the development of traditional reversal agents has predominantly relied on simplified resistance models based on the overexpression of a single ABC transporter. This approach exhibits significant discrepancies with the complex resistance microenvironments observed in clinical tumor patients. Clinical tumors frequently present with the synergistic co-expression of multiple ABC subtypes (e.g., ABCB1 + ABCC1) and multi-mechanism cross-resistance (transporter overexpression + aberrant apoptosis pathways). Single-target reversal agents struggle to address such intricate resistance scenarios. Furthermore, the absence of personalized treatment regimens based on patient-specific transporter expression profiles leads to suboptimal responses in some patients, further highlighting the limitations of their clinical relevance.

Currently, researchers are exploring the next generation of inhibitors, including compounds isolated from natural sources and their derivatives [73,74,75]. Because of the advantages of natural products such as cost and access, researchers have identified compounds synthesized with 7-isopentenyloxycoumarin (7-IP), chalcone and jatropha diterpenoids natural products as precursors. Most terpenoids originate from plants with dual medicinal and edible properties (such as ginsenosides in ginseng, limonene in dried tangerine peel, and glycyrrhetinic acid in liquorice root). These compounds exhibit low in vivo toxicity and excellent biocompatibility, thereby circumventing the drawbacks of traditional synthetic MDR reversal agents (e.g., verapamil), which are often rendered clinically impractical due to significant adverse effects:

However, current research on natural reversal agents still faces the following knowledge gaps:

(1) The mechanisms of action for most terpenoid reversal agents remain unclear, particularly regarding their selectivity for ABC transporter subtypes and their effects on signaling pathways.

(2) In vivo studies and pharmacokinetic/toxicological data are lacking.

(3) The synergistic effects of combining terpenoids with other chemotherapeutic agents and their potential for clinical translation require urgent exploration.

Furthermore, certain terpenoids have advanced to clinical or preclinical research stages (e.g., paclitaxel derivatives), possessing refined extraction, separation, and preparation technologies. This establishes a robust foundation for their clinical translation as MDR reversal agents. Consequently, we aim to provide insights for further reversal agent development by summarizing terpenoid compounds, examining their chemical structures, and synthesizing reversal mechanisms.

## 4. Progress of Terpenoids in the Study of Tumor MDR

### 4.1. Terpenoids: Chemical and Biosynthetic Overview

Terpenoids rank among nature’s most structurally diverse and widely occurring natural products. Their distinctive skeletal structures and diverse biological activities render them a focal point of research across pharmaceuticals, agriculture, and daily chemicals. Terpenoids are a class of natural organic compounds widely found in nature. They consist of isoprene units (C5H8), their molecular formulas have a simple multiplicative relationship with isoprene, and the general formula can be written as (C5H8) n. Terpenoids are often classified based on the number of isoprene units in the molecular skeleton, such as monoterpenes, sesquiterpenes, diterpenes, and triterpenes.

#### 4.1.1. Classification of Terpenes

Beyond the traditional classification based on the number of isoprene units, terpenoids can be categorized through multiple dimensions, including structural skeleton characteristics, functional group types, and biosynthetic pathways. This approach overcomes the limitations of a single classification system.

##### Classification by Structural Framework Complexity

Chain terpenes: This class of terpenoid compounds possesses an acyclic structure, featuring only linear arrangements of carbon–carbon single and double bonds within the molecule. For instance, compounds such as geraniol and farnesol are commonly found in plant essential oils, imparting a fresh, volatile fragrance to the plants [76,77]. Monocyclic terpenes: These possess a single carbon ring within their molecular structure, typically formed by the end-to-end coupling of isoprene units to close the ring. Common examples include the menthane-type menthone and the pinane-type *α*-pinene. These compounds frequently serve as core constituents in flavorings and natural antimicrobial agents [78,79]. For instance, limonene—a monoterpene commonly found in citrus fruits—possesses a pleasant citrus aroma and is extensively utilized in the flavoring industry [78].

Polycyclic terpenoids: These compounds contain two or more carbon rings, interconnected via fused, bridged or spiro rings to form more complex structures. The tetracyclic diterpene skeleton of paclitaxel and the pentacyclic triterpene structure of oleanolic acid are typical examples. These intricate ring systems constitute the fundamental structural basis for their distinctive pharmacological activities. For instance, paclitaxel’s complex ring architecture is crucial to its anti-microtubule action as a significant anti-cancer drug [80]

##### Classification by Functional Group Modification Type

Terpenes: This class of terpenoid compounds contains only carbon and hydrogen elements, with no other functional groups. Limonene and pinene are typical representatives of terpenes, representing the most fundamental forms of terpenoid compounds and often serving as primary constituents of plant essential oils [81]. For instance, metabolomic studies on kombucha models indicate terpenoids as one of their principal chemical components 13. Aromatic analysis of white tea also indicates that terpenoids (such as linalool and nerolidol) constitute significant components of its aroma profile [82]. In lovage, terpenoids including limonene and carvone are abundant and exhibit antibacterial and antifungal properties [83].

Oxygenated terpenoids: These terpenoid compounds incorporate oxygen-containing functional groups such as hydroxyl, carbonyl, carboxyl, or ester groups into their molecular structures. The introduction of these oxygen functional groups typically enhances the biological activity of terpenoid derivatives significantly. For instance, paeoniflorin is a monoterpenoid glycoside containing a glycosidic bond and exhibits anti-inflammatory activity; artemisinin, a sesquiterpenoid lactone featuring a peroxy bridge, possesses notable antitumor properties; while oleanolic acid, containing a carboxyl group, demonstrates hepatoprotective effects. Oxidative modifications of terpenoids, such as those mediated by unspecific peroxidases (UPOs), can generate a series of high-value oxygenated terpenoids with substantial potential for synthetic applications [84,85,86].

Nitrogen/Sulphur-Containing Terpenoids: These terpenoid derivatives are relatively uncommon, predominantly occurring in marine organisms and microorganisms [87]. For instance, nitrogen-containing sesquiterpene alkaloids isolated from sponges exhibit potent antiviral activity. These heteroatomic functional groups serve as the defining hallmark distinguishing nitrogen/sulphur-containing terpenoids from conventional terpenoids. For instance, glutathione is a sulphur-containing tripeptide involved in terpenoid metabolism and detoxification processes within organisms [88,89].

### 4.2. Monoterpene

Monoterpenes are compounds with a molecular skeleton consisting of two isoprene units and containing 10 carbon atoms. They are widely distributed in secretory tissues such as glands, oil chambers and resin tracts of higher plants and are the main constituents of plant volatile oils and are also present in metabolites of insects and microorganisms and marine organisms [90]. Compared to other compounds, monoterpenes MDR reversing agents are less common.

#### 4.2.1. Monoterpenes Directly Inhibits Drug Efflux Mediated by ABC Transporters

Carvacrol (**1**), the main monoterpene in Thymus plants, has been shown via molecular docking to form stable interactions within the P-gp drug-binding pocket. Its affinity (−7 kcal/mol), mediated by hydrophobic contacts (e.g., with Ser228, Ala229, Trp232) and a hydrogen bond (Gln990), is slightly lower than that of verapamil (−8 kcal/mol), yet it effectively inhibits P-gp function [91]. In MCF-7 breast cancer cells, carvacrol combined with 5-FU significantly increased intracellular 5-FU accumulation and enhanced apoptosis by 6.7-fold, outperforming the verapamil +5-FU combination (4.26-fold). ADME analysis indicated compliance with Lipinski’s rule, good gastrointestinal absorption, and low toxicity, supporting its drug-like potential.

Monoterpenes from Zanthoxyli Fructus, including (R)-(+)-citronellal and (S)-(−)-*β*-citronellol (**2**), also directly target P-gp. The ethyl acetate extract exhibited the strongest P-gp inhibition in LLC-GA5-COL150 cells (IC_50_ = 166 μg/mL), with individual IC_50_ values of 167 μM and 504 μM, respectively. These monoterpenes specifically increased intracellular accumulation of [^3^H] digoxin (a P-gp substrate) without affecting passive diffusion of [^3^H]3-O-methyl-D-glucose, confirming a direct P-gp inhibition mechanism rather than membrane disruption [92].

The *β*-cyclocitral derivative TMPE (**3**), a monoterpenoid analog, directly inhibits transport activity without altering ABC transporter expression. In LoVo/Dx and HT29/Dx resistant colon cancer cells, TMPE significantly enhanced intracellular accumulation of P-gp substrates (rhodamine 123, doxorubicin), while RT-PCR and Western blot showed no significant change in ABCB1 mRNA or protein levels [93]. Molecular simulations suggest that side-chain modifications enable TMPE to interact with non-substrate binding sites of P-gp, interfering with ATPase activity and blocking drug efflux. Notably, TMPE exhibited selective toxicity toward resistant cells with minimal effects on sensitive counterparts, highlighting its favorable safety profile.

#### 4.2.2. Modulation of Drug-Metabolizing Enzymes and Nuclear Receptor Signaling Pathways

One key mechanism of multidrug resistance (MDR) involves tumor cells accelerating the degradation of chemotherapeutic agents by upregulating drug-metabolizing enzymes. Monoterpenoid compounds (**4**) can interfere with drug inactivation processes by modulating metabolic enzymes and their upstream nuclear receptor signaling pathways. Lemongrass essential oil and its major component, citral, simultaneously target transporter and metabolic enzyme systems: co-administration with doxorubicin reduced the doxorubicin IC_50_ in MCF-7/ADR and HepG-2/ADR cells by 1.5–4.1-fold, exhibiting a synergistic effect (CI < 1). Mechanistically, this occurs by downregulating Phytoestrogen Receptor X (PXR) expression, thereby inhibiting transcription of downstream CYP3A4, GST, and ABC transporters, resulting in a dual increase in intracellular drug concentrations [94].

#### 4.2.3. Enhancing Drug Delivery Efficiency and Tissue Barrier Penetration

Moreover, refractory tumors (such as gliomas) characterized by poor blood–brain barrier permeability, coexisting multidrug resistance (MDR), and monoterpene components can enhance drug delivery efficiency. Borneol-modified docetaxel + aristolochic acid micelles (B-DTX-TET-M) (**5**) not only inhibit P-gp function but also modulate blood–brain barrier tight junctions, thereby increasing drug accumulation within the brain. This micelle significantly reduced the IC_50_ for U87/ADR cells and promoted apoptosis in drug-resistant cells (downregulating Bcl-2 and upregulating Bax/caspase-3). In vivo experiments demonstrated its specific accumulation at glioma sites, achieving a tumor inhibition rate of 78.3% [95].

In summary, the structure–activity relationship (SAR) of monoterpenoid compounds exhibits distinct structural dependencies: cyclic skeletons (e.g., carvacrol, borneol) leverage their rigid structures to fit P-glycoprotein (P-gp) binding pockets, inhibiting drug efflux via hydrophobic interactions and hydrogen bonding, while chain-like skeletons and derivatives (e.g., citral, TMPE) utilize structural flexibility to achieve dual regulation of transporters and metabolic enzymes. Polar functional groups (hydroxyl, carbonyl) and hydrophobic substituents (methyl, isopropyl) enhance target binding affinity, while conjugated double bonds boost molecular activity. Stereochemistry directly determines action specificity, exemplified by (R)-(+)-citronellal exhibiting markedly superior inhibitory activity compared to its isomers.

**Figure 3:**
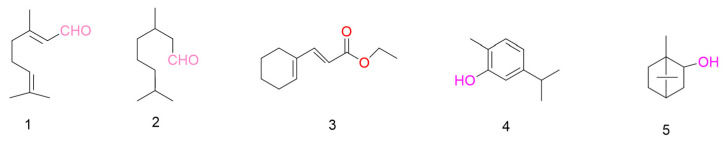
Structures of monoterpene compounds.

The selected lead compounds each possess distinct advantages: Apigenin, through an optimal combination of its monocyclic structure and functional groups, exhibits high affinity binding to P-glycoprotein (P-gp) and significantly enhances the apoptosis-inducing effects of chemotherapeutic agents, demonstrating outstanding drug potential. Citral downregulates PXR and drug resistance-associated gene expression, exhibiting synergistic chemotherapeutic activity against multiple drug-resistant cell lines; TMPE, following structural modification, exhibits selective toxicity towards drug-resistant cells with low off-target risk; Borneol combines blood–brain barrier penetration with P-gp inhibition, offering a novel approach for brain-resistant tumors; and (R)-(+)-Citronellaldehyde inhibits P-gp with high specificity without compromising cell membrane integrity. These compounds lay the foundation for subsequent structural optimization and clinical translation. The structures and detailed mechanisms of the above compounds are presented in Figure 3 and Table 2.

### 4.3. Sesquiterpenes

Sesquiterpenes are a class of naturally occurring terpenes with 15 carbon atoms in their molecules. They are formed by the polymerization of three isoprene units, and most sesquiterpenes conform to the isoprene rule. Sesquiterpenoids are widely distributed in nature, especially abundant in plants of the *Magnoliaceae*, *Rutaceae*, *Cornaceae*, and *Chrysanthemum* families. In plants, sesquiterpenes often occur in volatile oils like alcohols, ketones, lactones, etc., and are the main components of the high-boiling portion of the volatile oil.

#### 4.3.1. Competitive Inhibition of ABCB1 (P-gp) Transport Function

This mechanism represents the core pathway by which sesquiterpenoids reverse multidrug resistance (MDR). By competing with chemotherapeutic agents for binding to the drug-binding pocket of P-glycoprotein (P-gp), they block its ATP-dependent efflux function, thereby elevating intracellular drug concentrations, For example, the sesquiterpene lactone compound Conferone competitively inhibits P-gp in MDCK-MDR1 cells, whereas [^125^I] IAAP for binding sites (Ki = 6.2 μM), reducing the IC_50_ of vincristine from 12.8 μM to 2.3 μM—a 5.6-fold reversal—while also increasing the intracellular accumulation of rhodamine 123 in a dose-dependent manner (IC_50_ = 8.7 μM) [96]. The limonoid sesquiterpene obacunone, acting via the same mechanism, exhibited ED_50_ values of 0.028 μg/mL and 0.0011 μg/mL in P-gp-overexpressing MES-SA/DX5 and HCT15 cells, respectively, demonstrating activity comparable to the classical P-gp inhibitor verapamil. Crucially, it exhibits no toxicity in non-resistant cells (IC_50_ > 30 μg/mL), thereby avoiding off-target effects. A shared characteristic of this class of compounds is their precise targeting of P-gp’s substrate-binding region. They do not interfere with P-gp’s mRNA or protein expression, instead selectively inhibiting its transport function [97]. Dihydro-*β*-agarofuran sesquiterpene specifically binds to the P-gp homolog protein in Leishmania parasites. At a concentration of 10 μM, it reduces the IC_50_ of metifoxine in drug-resistant strains by 4–7-fold, while exhibiting low cross-reactivity with mammalian ABCB1. Its mechanism involves acting on the transmembrane domain of the transporter to block efflux via mixed-type inhibition [98].

#### 4.3.2. Subtype-Selective Targeting of ABC Transporters

In P-gp-overexpressing MCF-7/ADR cells, low-polarity sesquiterpene lactone compounds exhibited stronger reversal activity, achieving a reversal factor of 12.26. In contrast, within ABCC1 (MRP)-overexpressing Bel7402/5-FU cells, highly hydrophilic sesquiterpene lactone compounds (e.g., compound 12, ASA-P = 200) demonstrated superior efficacy, achieving an 8.63-fold reversal ratio. Their hydrophilic structure facilitates interaction with MRP’s preferred anionic drug binding sites. The sesquiterpene compound *β*-caryophyllene, targeting solely ABCB1 with negligible inhibition of ABCC1 or ABCG2, further demonstrates subtype selectivity advantages, potentially reducing interference with physiological transport functions in normal tissues [99].

The configuration of monoterpenes is a key determinant in modulating their activity against tumor multidrug resistance (MDR), exhibiting clear structure–activity relationships. The skeleton type governs target specificity toward ABC transportersmenthane” and “pinane-type” frameworks, typically containing six-membered or oxygen-heterocyclic rings, interact with the drug-binding pockets of transporters such as ABCB1 (P-gp) through hydrophobic contacts and hydrogen bonds, whereas ‘geraniane-type’ conjugated structures facilitate π-π stacking with protein residues. Substituent position, configuration, and type directly influence potency—hydroxyl groups at C-3 and C-6 enhance hydrogen-bonding capacity, while alkyl groups at C-2 and C-7 optimize lipophilicity (logP_o_/w ≈ 3.5–5.0) to promote membrane permeation; *α*-configured substituents generally form more stable interactions with binding pockets. Physicochemical compatibility further dictates subtype selectivity: low-polarity monoterpenes tend to target ABCB1-mediated resistance, while more hydrophilic analogs are better suited for ABCC1 (MRP)-associated MDR, enabling precise intervention against different resistance mechanisms.

**Figure 4:**
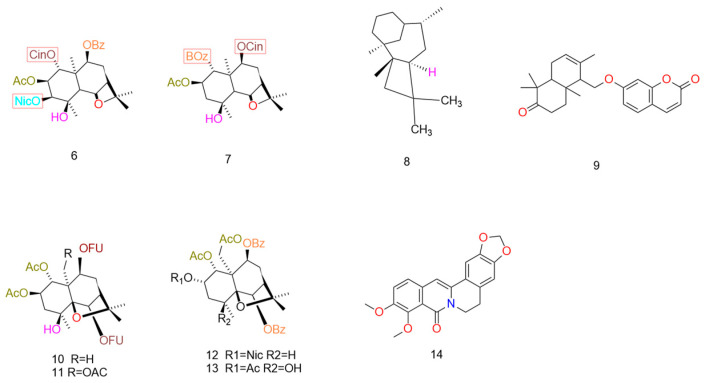
Structures of sesquiterpene compounds.

Based on advantages in configuration, potency, and draggability potential, the lead compounds are prioritized as follows: Dihydro-*β*-agarofuran-type sesquiterpenoids are assigned the highest priority due to their novel tetracyclic skeleton, which confers species-selective targeting—effectively addressing the gap in therapies against multidrug-resistant (MDR) parasitic infections—along with low toxicity and minimal cross-reactivity. Conferone, representing the sesquiterpene lactone class, ranks second, given its well-defined mechanism as a competitive inhibitor of ABCB1, coupled with a readily modifiable structure that enhances its drug development outlook. *β*-Caryophyllene is positioned third, supported by its dual mechanisms of action, leading to strong synergistic effects, wide natural availability, and a well-established safety profile, making it suitable for accelerated preclinical advancement. Obacunone, though equipotent to verapamil in activity, is ranked fourth due to challenges in modifying its rigid scaffold, poor aqueous solubility, and the consequent need to overcome key draggability barriers. Finally, 1H-Indole-3-carbaldehyde is placed fifth, as its potent bioactivity is offset by metabolic instability associated with the indole ring, likely extending its development timeline. The structure and mechanism of the sesquiterpenes are presented in Figure 4 and Table 3.

### 4.4. Diterpenoids

Diterpenoid compounds are comparatively more abundant than monoterpenoids. This paper analyses the configurations of 64 compounds and their mechanisms for reversing tumor multidrug resistance (MDR) across 29 literature sources. The mechanisms by which diterpenoids reverse tumor multidrug resistance can be categorized into six core types. The primary mechanism, documented in 23 publications, is “direct inhibition of P-gp function”. This is followed by “dual/multiple target inhibition” and “regulation of resistance-associated signaling pathways”.

#### 4.4.1. Diterpenoid Compounds Directly Inhibit P-gp Function

Direct P-gp targeting and efflux inhibition are the primary MDR reversal mechanisms of diterpenoids, where compounds interfere with chemotherapeutic efflux via pocket binding or channel blocking (without affecting P-gp expression) and feature direct action and rapid efficacy.

The drug-binding pocket of P-gp comprises multiple critical amino acid residues, where diterpenoid compounds may interact with residues such as Ser725, Phe728, and Arg905 through intermolecular forces including hydrophobic interactions and hydrogen bonds. This enables competitive inhibition of the binding between chemotherapeutic agents and P-gp [101,102]. For example, jatrophane-type diterpenoids derived from *Euphorbia glomerulans* exhibit a binding affinity for P-gp of −8.4 kcal/mol, significantly outperforming the positive control verapamil (−7.5 kcal/mol); with a half-maximal effective concentration (EC_50_) as low as 159.5 nM, demonstrating a dose-dependent enhancement of doxorubicin (DOX) and rhodamine 123 (Rh123) accumulation within MCF-7/ADR cells [101]. Additionally, 6*β*,7*β*-epoxy-3*β*,4*β*,5*β*-trihydroxy-20-deoxyingenanol (ingenane type) derived from *Euphorbia kansui* exhibited a reversal factor (RF) of 186.4 against multidrug resistance, significantly higher than verapamil (RF = 93.7), and demonstrated no apparent cytotoxicity in HepG-2/Adr cells (IC_50_ > 40 μM) [103].

**Figure 5:**
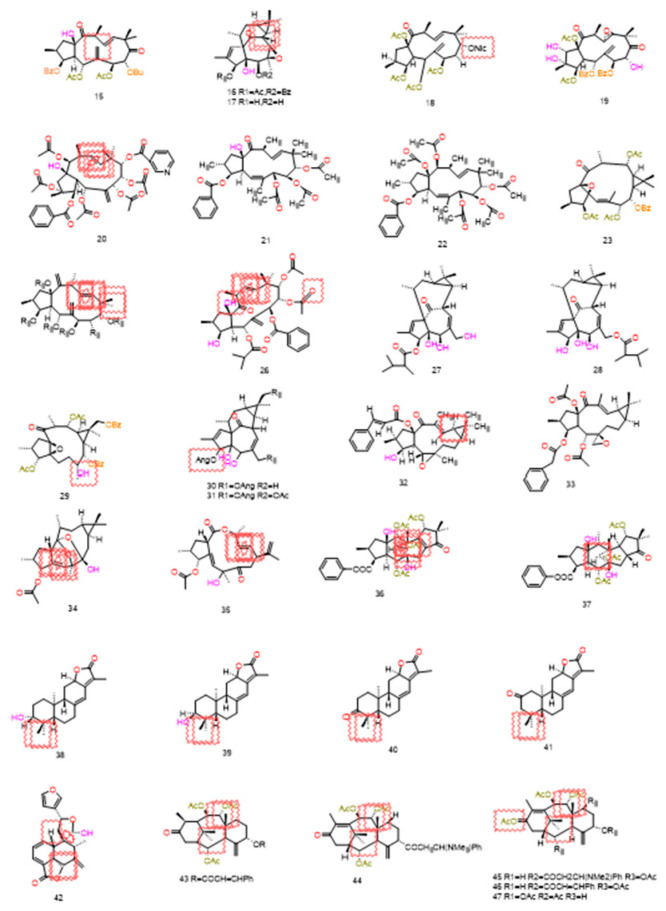
Structures of diterpenoids compounds.

Certain diterpenoid compounds indirectly impair drug efflux by inhibiting P-gp’s ATPase activity, thereby disrupting its energy supply. For instance, treatment with jatrophane-type compounds derived from *Euphorbia glomerulans* reduced P-gp ATPase activity by up to 67%. Concurrently, these compounds induce reactive oxygen species (ROS) accumulation within drug-resistant cells, further amplifying DOX-induced apoptosis [101]. Moreover, Helioscopinolide F, belonging to the Ent-abietane type, exhibited a fluorescence activity ratio (R) as high as 138.56 at a concentration of 40 μg/mL, significantly outperforming verapamil (R = 12.23). Furthermore, it effectively inhibited the efflux of Rh123 within a short treatment period (30–35 min), demonstrating a rapid onset of action [104]. Table 4 and Figure 5 summarize all diterpene compounds that directly inhibit P-gp function and their primary configurations.

#### 4.4.2. Dual-Target/Multi-Target Inhibition

**Figure 6:**
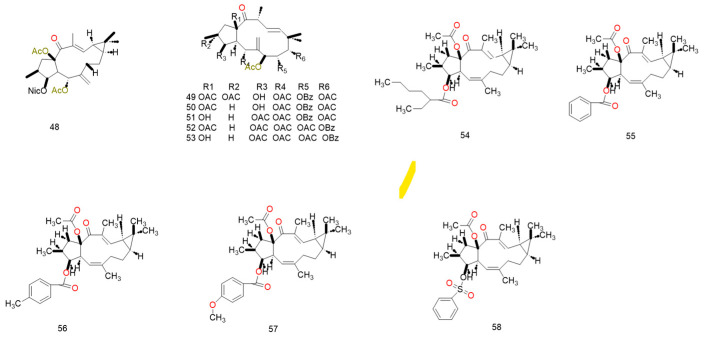
Structures of diterpenoids compounds.

Certain diterpenoid compounds exhibit P-gp-specific inhibitory activity without affecting the function of other ABC transporters, displaying characteristics similar to third-generation multidrug resistance (MDR) inverters [105]. Lathyrane-type compounds (**48**) derived from *Euphorbia lathyris* simultaneously inhibit P-gp and MRP1, significantly reducing the mRNA levels of ABCB1 and ABCC1 in HepG2-ADR cells, thereby elevating DOX-induced apoptosis to 42.3% [112]. Jolkinol D (**58**) derivatives not only inhibit the efflux function of P-gp and MRP1 (RF = 38.2) but also reduce the membrane localization levels of both transporters (by 53% and 47%, respectively). This dual mechanism of “functional inhibition and localization disruption” enhances the reversal effect [117]. These studies indicate that dual target/multi-target diterpene compounds possess more comprehensive potential for reversing drug resistance. The structures and mechanisms of the above compounds are presented in Figure 6 and Table 5.

#### 4.4.3. Other Relevant Mechanisms

Regulating P-gp expression at the transcriptional or translational level to reduce transporter numbers at the source represents another crucial mechanism by which diterpenoid compounds reverse multidrug resistance. These compounds primarily achieve this by inhibiting MDR1 gene transcription, diminishing promoter activity, or reducing protein synthesis, thereby progressively weakening tumor cells’ resistance capacity over time.

Compounds of the jatrophane type derived from *Euphorbia kansui* inhibit the activity of the MDR1 gene promoter, reducing P-gp protein levels by 58%. Their IC_50_ value is 2.3 μM, with a relative frequency (RF) of 32.1 [85].

**Figure 7:**
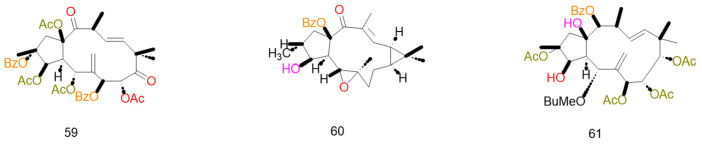
Structures of diterpenoids compounds.

The activation of P-gp ATPase activity represents a unique MDR reversal mechanism specific to diterpenoid compounds, with few published reports to date. Such compounds indirectly inhibit P-gp’s drug transport function by interfering with its energy metabolism cycle, without altering P-gp expression levels, constituting a “functional interference” mechanism. Compound (**61**) a jatrophane-type compound derived from *Euphorbia sororia*, exhibits concentration-dependent activation of P-gp ATPase activity with an EC_50_ of 1.82 μM and a relative frequency (RF) of 26.90 [87]. It disrupts the energy balance of drug efflux by activating ATPase activity, thereby promoting the accumulation of Rh123 and DOX within MCF-7/ADR cells. Although this mechanism has been reported infrequently, it offers a novel perspective on the mode of action of MDR reversal agents, particularly for tumor models exhibiting resistance to conventional P-gp inhibitors. The structures and data of compounds sharing the same skeleton and mechanism are presented in Figure 7 and Table 6.

Diterpenoid compounds may indirectly reverse multidrug resistance (MDR) by modulating resistance-associated signaling pathways such as PI3K/Akt, MAPK, and Nrf2/ARE (addressed in five studies) and synergize with direct transport protein inhibition by downregulating resistance gene expression or enhancing chemotherapy-induced apoptosis to amplify reversal efficacy.

Abnormal activation of the PI3K/Akt pathway is closely associated with P-gp overexpression. Lathyrane-type compounds (**48**) derived from *Euphorbia* can inhibit this pathway, thereby enhancing DOX-induced apoptosis rates [118]. Moreover, diterpene compounds derived from *Salvia miltiorrhiza* downregulate the mRNA levels of the anti-apoptotic gene Bcl-2 (by 71%), thereby promoting apoptosis in drug-resistant cells. Their IC_50_ value is 3.1 μM, with a relative potency (RF) of 25.8 [89].

Certain diterpenoid compounds can directly induce apoptosis or arrest the cell cycle in drug-resistant cells and synergize with chemotherapeutic drugs by activating apoptotic pathways, damaging mitochondria, or disrupting cell cycle progression, and are thus particularly suitable for treating apoptosis-resistant tumors.

Latilaga scene B (**32**) activates the apoptotic pathway in mouse lymphoma cells while promoting the accumulation of Rh123 and DOX within LoVo/Dx cells. At a concentration of 100 μM, the fluorescence intensity ratio (FIR) reached 6.51 [113]. It is noteworthy that most such compounds do not induce apoptosis in normal cells, demonstrating a degree of tumor specificity [113,119]. All of the above compounds are listed in Table 4.

**Figure 8:**
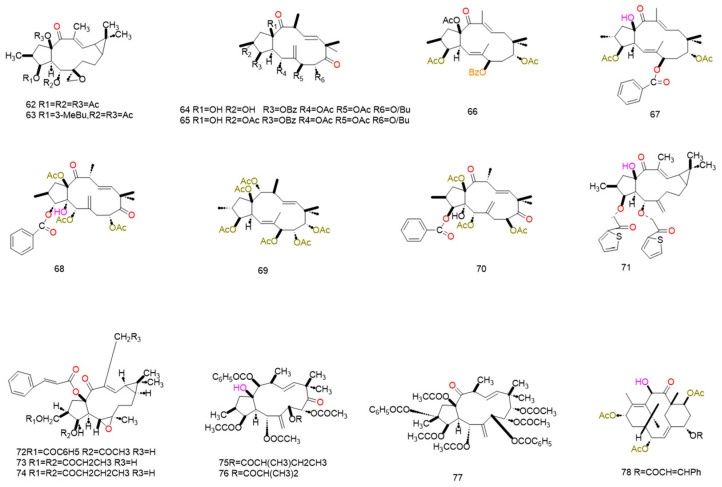
Structures of diterpenoids compounds.

Certain diterpenoids reverse cancer MDR via multidimensional synergistic effects. Jatrophane derivatives at 10 μM inhibit MDR1 (P-gp) and MRP (ABCC1) with 78% and 65% reversal rates, respectively, without regulating Bcl-2/Bax and other cytotoxic proteins, thus avoiding off-target risks [120]. Their complementary mechanisms—transporter inhibition, gene modulation, targeted binding, and chemosensitization—offer multifaceted therapies for drug-resistant tumors. Compounds sharing the same mechanism and skeleton are presented in Figure 8 and Table 7.

The structure–activity relationship (SAR) of MDR-reversing diterpenoids shows distinct structure-dependent features, providing a theoretical basis for rational design and optimization of potent reversal agents. Substituent type and position are core activity regulators: for jatrophane-type diterpenes, C-8 substituent activity follows -OiBu > -OBz > -OH ≈ H, with acyloxy substitutions at C-5/7/8 being essential; for ingenane-type diterpenes, the 6*β*,7*β*-epoxy moiety markedly enhances P-gp inhibition (e.g., 6*β*,7*β*-epoxy-3*β*,4*β*,5*β*-trihydroxy-20-deoxy-jatrophol). Additionally, the free C-3 hydroxyl boosts P-gp inhibition (abolished by acetylation), while the C-15 hydroxyl strengthens hydrogen bonding with P-gp’s active pocket to optimize binding. At the ring system level, the 12-membered macrocyclic flexibility of jatrophanes is a prerequisite for P-gp conformational matching, whereas segetane tetracyclic skeletons require strict maintenance of specific conformationsΔ^6^(^17^)/Δ^11^(^12^) double bonds yield optimal activity). Lipophilicity-wise, compounds with logP 4–6 balance membrane permeability and target affinity, and moderate substituent lipophilicity reduces cytotoxicity. Ranked by the three-dimensional “activity potency-target selectivity-drugability” system, first-tier lead compounds (ingenane-type: RF = 186.4; jatrophane-type: RF = 143.8) have high clinical translation potential; second-tier compounds need structural modification to improve water solubility; third-tier compounds can serve as combination chemotherapy adjuvants or P-gp functional study tool molecules.

Building on the above SAR patterns and lead compound structural features, future research should focus on three core directions: (1) deepen investigations into the SAR between jatrophane-type diterpenes and P-gp functional inhibition, systematically clarify the flexible traits of their 12-membered macrocyclic skeletons, the electronic/steric effects of substituents at key sites, and the stereochemistry-P-gp active pocket docking mechanism to unravel the molecular regulatory details of SAR correlations; (2) perform targeted structural optimization of highly active leads based on established SAR, including precise modulation of C-3/C-8 substituents, introduction of epoxy moieties to boost activity, double bond conformation tuning, and concurrent improvement of water solubility, in vivo stability, and tumor targeting; and (3) explore natural product structural diversity and synthetic modifications of privileged skeletons (e.g., jatrophane-type) to develop novel MDR reversal agents with high activity, target specificity, and favorable pharmacokinetics, thereby expediting their bench-to-clinic translation.

### 4.5. Triterpenes

Triterpenoids are widely distributed in plants, fungi and marine organisms, exhibiting diverse biological activities including anti-inflammatory, anti-tumor and immunomodulatory effects [123]. Recent studies have revealed that such compounds can reverse multidrug resistance (MDR) through mechanisms including direct inhibition of ABC transporter function and regulation of drug resistance-associated signaling pathways. Furthermore, the structural framework derived from natural sources provides a sound foundation for structural modification and optimization of drug-like properties [124,125]. This paper systematically reviews the mechanisms and structure–activity relationships of triterpenoid compounds in reversing multidrug resistance (MDR) based on high-quality research literature published in recent years. It establishes a scientifically grounded prioritization evaluation system to guide preclinical development

#### 4.5.1. The Direct Inhibition of ABC Transporter Function by Triterpenes and Its Structure–Activity Relationship

Tricarboxylic acid derivatives compete with chemotherapeutics for binding to this pocket, thereby inhibiting transporter-mediated drug recognition and efflux and elevating intracellular drug levels. For example, the 23-hydroxybetulinic acid derivative BBA (**84**) binds to a site partially overlapping ABCB1’s drug-binding pocket; it blocks the efflux of substrates (e.g., doxorubicin, rhodamine 123) via steric hindrance without altering ABCB1 mRNA transcription or protein expression [125]. Spiropedroxodiol—a spiro-triterpene isolated from *Euphorbiaceae* plants—induces dose-dependent accumulation of rhodamine 123 in L5178Y-MDR cells. At 20 μM, its fluorescence activity ratio (FAR) reached 96.02, markedly superior to the positive control verapamil (FAR = 9.66). This compound acts by directly binding to the ABCB1 modulator-binding site (M-site), stabilizing the protein conformation through π-π stacking and CH-π interactions to suppress transporter-mediated drug efflux [124].

#### 4.5.2. Other Mechanisms by Which Triterpenes Reverse Tumor Multidrug Resistance

Triterpenoids can reverse multidrug resistance (MDR) by targeting upstream signaling networks of drug resistance, with the specific intervention of the melianodol-type triterpenoid 21*α*-methyl melianodiol on the PI3K/mTOR pathway serving as a prime example. Abnormal activation of the PI3K/mTOR pathway upregulates ABCB1 (MDR1) transcription, enhances drug efflux, and promotes the synthesis of anti-apoptotic proteins, forming a dual resistance barrier. This compound competitively binds the ATP-binding site of PI3K kinase, inhibiting its phosphorylation activation, thereby blocking downstream mTOR signaling. This ultimately downregulates ABCB1 transcription levels and diminishes the efflux capacity of drug-resistant cells [126].

Beyond their core mechanism of action, triterpenoids exert MDR reversal effects via multiple auxiliary pathways. Representative compounds include alisol-type triterpenes (Alisol B 23-acetate, Alisol A 24-acetate) and oleanane-type glycyrrhetinic acid [8,11]. Alisolic acid-type triterpenes primarily regulate cell membrane properties and oxidative stress: they enhance membrane fluidity in drug-resistant cells, reducing the membrane adaptability necessary for drug efflux while promoting transmembrane uptake of chemotherapeutics to increase intracellular drug accumulation; additionally, they induce reactive oxygen species (ROS) elevation in resistant cells, disrupting redox balance, activating apoptotic signaling pathways, and amplifying chemotherapeutic cytotoxicity [127]. Experiments show that at non-cytotoxic concentrations, these compounds significantly improve membrane fluidity and intracellular drug accumulation in HepG2/Vin cells, upregulate ROS-mediated apoptosis-related proteins, and exert prominent synergistic effects with chemotherapeutics.

**Figure 9:**
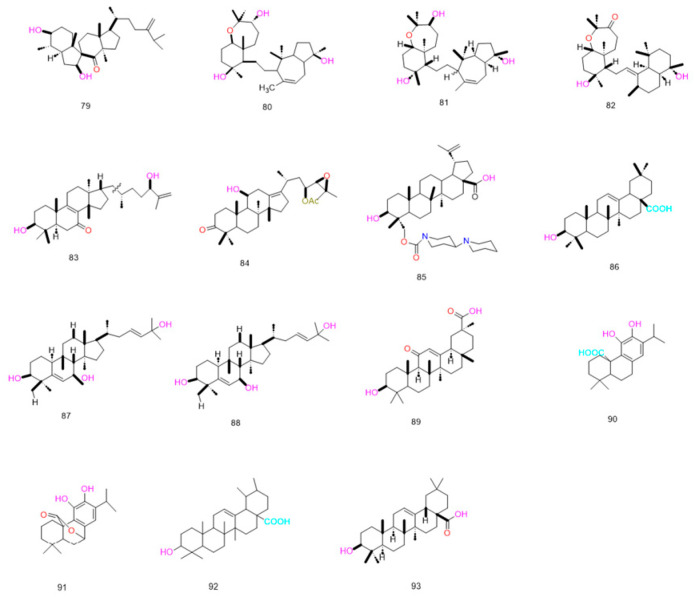
Structure of triterpene compounds.

Iquiritigenin acid exerts its adjuvant effect through a dual pathway of “functional inhibition and apoptosis regulation”. It can mildly suppress the expression and efflux function of ABCB1 while simultaneously modulating the expression of apoptosis-related factors (such as Bcl-2 family proteins), thereby reversing the apoptotic escape phenotype of drug-resistant cells and enhancing the cytotoxicity of chemotherapeutic agents [128]. Whilst not predominant, such auxiliary mechanisms complement core approaches targeting transporters directly or regulating signaling pathways. They prove particularly suited to complex MDR phenotypes mediated by multiple factors—including altered membrane properties, oxidative stress tolerance, and evasion of apoptosis. This not only broadens the application scope of triterpenoid compounds but also offers fresh academic perspectives for multi-mechanism combined reversal of MDR. All the above compounds are presented in Figure 9 and Table 8.

Triterpenoids exhibit structural diversity, with specific structural features critical for multidrug resistance (MDR) reversal activity. For example, hydroxyl groups at C-4 and C-7 of compound (**81**) aglycone (isolated from Red Sea sponge *Callyspongia siphonella*) are essential; their absence or modification significantly diminishes MDR reversal capacity. Compound (**82**) shows 1/8 the activity of cefuroxime A due to C-11 carbonyl substitution, while cefuroxime C (3) and I (4) exhibit only 2–3-fold reversal effects (much lower than cefuroxime A) due to deviations from the typical cefuran skeleton (e.g., cefurane skeleton in 3, 14,15-epoxy group in 4). The lanostane-type triterpene ABA requires a “tetracyclic skeleton” (cis-fused A/B rings, C-11 hydroxyls, C-3 carbonyl) for activity: the tetracyclic planar aromatic structure binds P-gp hydrophobic sites and C-11 hydroxyl/C-23 acetate to form key hydrogen bond networks. The C-23 acetate is indispensable (hydrolysis to hydroxyl reduces lipophilicity, impairing membrane penetration and P-gp binding), and the C-3 carbonyl is essential (reduction to hydroxyl weakens π-π interaction with P-gp, decreasing MDR reversal activity by over 50%).

In summary, common structural determinants of triterpenoid MDR reversal activity include the following: (1) Rigid parent skeleton topology: triterpenes with rigid polycyclic frameworks (e.g., xypholane bicyclic, spirocyclic triterpene spirocarbon, lanosterol tetracyclic) better match the P-gp substrate-binding pocket, showing higher activity than flexible-skeleton analogs. (2) Hydroxyl/carbonyl positions and quantity: 3*β*, 7*β*, 11*α* hydroxyls and C-3 carbonyl are highly active (forming hydrogen bonds with P-gp Ser/Thr residues to enhance affinity), while excess hydroxyls reduce activity via steric hindrance. (3) Hydrophobicity and aromaticity: moderate lipophilicity (logP = 3–5) ensures membrane permeability; aromatic rings (e.g., benzoic acid, furan) enhance π-π interactions with P-gp aromatic residues, boosting activity. (4) Side-chain configuration and integrity: chiral centers (e.g., C-24 R configuration) and intact carbon chains are critical; side-chain oxidation, cleavage, or configuration inversion markedly reduces activity.

## 5. Conclusions

Multidrug resistance (MDR) in tumors is the primary cause of chemotherapy failure. This review systematically elucidates how terpenoids, with their remarkable structural diversity, constitute a treasure trove of natural compounds for overcoming MDR. They reverse resistance through multi-targeted, multi-level synergistic actions—including direct inhibition of ABC transporter function, downregulation of their expression, and modulation of cell death pathways such as apoptosis or autophagy—offering unique advantages beyond single-target inhibitors.

### 5.1. Comparative Analysis of Terpenoid Subtypes and Lead Compound Screening

Following a systematic comparative analysis of terpenoid subclasses, their pharmaceutical development potential and existing challenges exhibit a distinct gradient pattern: triterpenoids (exemplified by botulinic acid and oleanolic acid) currently represent the most valuable source of lead compounds for development [136]. This class of compounds possesses a stable chemical structure, and their multi-target activity has been conclusively validated. They can simultaneously regulate drug transporters such as P-gp and MRP1, as well as the PI3K/Akt signaling pathway, while exhibiting high in vitro activity. This multidimensional action is closely linked to their diverse functional groups and stereochemical configurations, making structure–activity relationship studies a critical scientific challenge in this field, and it serves as an excellent candidate template for the physical design of targeted drugs [137,138]. The reversal of tumor multidrug resistance (MDR) by diterpenoids exhibits diversity and synergistic mechanisms, with direct inhibition of P-glycoprotein (P-gp) function serving as the core mode of action, while dual/multi-target inhibition and signaling pathway regulation offer novel intervention strategies against complex resistance models. Structure–activity relationship (SAR) studies indicate that the activity of these compounds is predominantly influenced by the following structural factors: the type of substituents (e.g., isobutanoyloxy at C-8), ring system characteristics (e.g., the 6*β*,7*β*-epoxy fragment), and lipophilicity parameters (logP values in the range of 4–6) [139]. The jatrophane-type diterpenoids demonstrate potent P-gp binding affinity and effective MDR reversal activity, attributable to the structural flexibility of their 12-membered macrocyclic skeleton, diverse substituent modifications (e.g., an isobutanoyloxy group at C-8 and a free hydroxyl group at C-3), and suitable lipophilicity (logP ≈ 4–6) [140]. Monoterpenes and sesquiterpenes (such as *β*-elemene and artemisinin derivatives) possess synergistic potential in combination therapies due to their low molecular weight and excellent membrane permeability. For instance, *β*-elemene not only interferes with P-gp-mediated efflux but also activates apoptotic pathways and downregulates anti-apoptotic proteins such as Bcl-2, thereby synergistically enhancing the cytotoxicity of drugs like cisplatin and doxorubicin. However, the potency of such monotherapy components is relatively low, making them more suitable as adjuvant enhancers within combination treatment regimens rather than as core therapeutic agents [141,142].

#### 5.1.1. The Precise Structure-Mechanism-Activity Relationship Is the Core Principle Underlying Terpenoids-Mediated MDR Reversal

The skeleton types, substituent characteristics (e.g., C-3/C-23 hydroxyl groups, 7*β*-methoxy group), and physicochemical properties (optimal logP = 3.5–5.8) of triterpenoids (such as lupane-type BBA and cucurbitane-type balsaminol C) and sesquiterpenoids (such as lactone-type Conferone and dihydro-*β*-agarofuran-type compounds) directly determine their targeting specificity towards ABC transporters (primarily ABCB1, with partial targeting of ABCC1/ABCG2). Most terpenoids exert their effects through competitive inhibition of drug efflux, subtype-selective binding, or synergistic activation of apoptotic pathways, forming a clear structure–mechanism–activity correlation.

#### 5.1.2. Terpenoids Possess Irreplaceable Advantages over Traditional MDR Reversers

Compared with the first three generations of synthetic reversers, natural/semisynthetic terpenoids integrate low toxicity (mostly derived from edible medicinal plants, IC_50_ > 50 μM), high target specificity (e.g., oleanolic acid specifically inhibits ABCC1 without cross-reactivity with ABCB1), and multi-mechanism synergy (e.g., *β*-caryophyllene mediates “efflux inhibition + apoptosis activation”). These characteristics enable terpenoids to effectively circumvent the limitations of traditional drugs, such as high toxicity and drug–drug interactions.

#### 5.1.3. Multiple Classes of Terpenoids Exhibit Distinct Clinical Translation Potential

Among triterpenoids, lupane-type BBA and euphane-type sooneuphanone D, as well as sesquiterpenoids including dihydro-*β*-agarofuran-type compounds and Conferone, have emerged as the most promising lead compounds due to their potent in vitro and in vivo MDR reversal activity, low toxicity, and druggability for structural modification. They are particularly suitable for ABCB1-mediated tumor MDR and parasite MDR scenarios. Among these, a lupane-type BBA enhanced the inhibitory effect of doxorubicin against drug-resistant tumors by 53.6-fold in a nude mouse xenograft model, and no significant toxicity was observed (IC_50_ > 50 μM). Additionally, dihydro-*β*-agarofuran-type compounds reduced the IC_50_ of metronidazole by 4- to 7-fold in Leishmania-resistant models. These findings support the clinical translation potential of these compounds, although further large-animal studies and pharmacokinetic research are still necessary.

#### 5.1.4. Structural Modification and Precise Targeting Are Key Strategies to Improve Draggability

The activity of terpenoids depends on specific configurations (e.g., C-17 side chain of triterpenoids, lactone ring of sesquiterpenoids). Optimizing water solubility and metabolic stability through modifications such as glycosylation and ureidation or implementing individualized combination therapy based on transporter subtype expression profiles can significantly enhance the success rate of clinical translation.

### 5.2. Clinical Translation Potential and Practical Challenges of Terpenoid Multidrug Resistance Reversal Agents

Numerous natural products or their derivatives have demonstrated significant multidrug resistance reversal effects in in vitro cellular experiments. However, most research remains confined to the in vitro stage and focuses on single targets (predominantly P-gp). Two core bottlenecks that impede the clinical translation of natural product-derived multidrug resistance (MDR) reversers are poor lipophilicity and high intrinsic toxicity. To address these issues, a synergistic strategy incorporating structural modification, advanced nanodelivery systems, multi-target design, and pharmacokinetic optimization has been validated and established. As a preferred approach for improving druggability, structural modification can enhance membrane permeability by introducing lipophilic moieties at inactive sites, mitigate off-target toxicity through selective structural modification, and achieve targeted drug release via the construction of tumor microenvironment-responsive prodrugs [143,144,145]. Advanced nanodelivery systems (e.g., liposomes, PLGA nanoparticles, micelles) effectively improve drug solubility, enable tumor-targeted delivery via surface modification, and mitigate peak concentration-related toxicity through sustained release. Multi-target strategies (natural product combination, hybrid molecule design) synergistically inhibit multiple ABC transporters and their associated signaling pathways, lowering the effective doses of individual agents to achieve toxicity reduction and efficacy enhancement. Additionally, pharmacokinetic optimization circumvents enzyme system interference, reduces drug–drug interactions, and alleviates systemic toxicity accumulation. Collectively, these integrated strategies efficiently reconcile the trade-off among lipophilicity, efficacy, and toxicity of natural product-derived MDR reversers, thereby providing robust technical support for their clinical translation [146,147]. To date, the efficacy of most terpenoid compounds in reversing resistance has been validated in vitro through cellular experiments. However, in vivo studies remain largely confined to mouse xenograft models, lacking validation data from large animal studies. Furthermore, no compound has yet progressed to Phase III clinical trials, indicating that numerous unresolved issues persist regarding their clinical translation.

Terpenoids are predominantly derived from medicinal and edible homologous plants (e.g., ginsenosides from ginseng, glycyrrhetinic acid from licorice, cucurbitane-type triterpenoids from bitter melon) and have evolved favorable in vivo tolerance. Preclinical evidence demonstrates their extremely low toxicity against normal cell lines (e.g., NIH/3T3, MRC-5), with IC_50_ values mostly exceeding 50 μM, which potentially circumvents the severe toxicities (e.g., cardiovascular suppression, neurotoxicity) associated with first-generation MDR reversers such as verapamil and tariquidar. Additionally, terpenoids exhibit subtype-selective inhibitory activity against ATP-binding cassette (ABC) transporters: oleanolic acid specifically targets ABCC1 without cross-reactivity to ABCB1, while betulinic acid preferentially inhibits ABCB1. This selectivity enables precise suppression of abnormally overexpressed ABC transporters in tumor cells while sparing physiologically essential ABC subtypes in normal tissues (e.g., intestinal ABCG2, hepatic ABCC2), thereby mitigating the risk of systemic adverse effects.

Notably, the structural scaffolds of terpenoids (e.g., lupane-type, cucurbitane-type triterpenoids) and their key modifiable moieties (e.g., C-3/C-23 hydroxyls, C-28 carboxyl) are amenable to structural optimization via glycosylation, acylation, or hybrid modification strategies. Such modifications effectively enhance aqueous solubility, metabolic stability, and targeting capacity, addressing the structural optimization bottlenecks commonly faced by traditional synthetic MDR reversers.

### 5.3. Future Development Prospects

First, enhanced mechanism research: Multi-omics-driven in vivo action network analysis overcomes the limitations of traditional single-cell line in vitro screening. Integrating chemical proteomics and spatial transcriptomics, this approach systematically delineates the in vivo target profiles and signals regulatory networks of terpenoids within the tumor microenvironment, enabling a paradigm shift from single-target validation to multi-pathway synergistic mechanism elucidation.

Second, technology-driven formulation innovation: Moving beyond the traditional active ingredient-centric screening paradigm, research now prioritizes structure-based modification and advanced delivery system development. Liposomal encapsulation, exosome-mediated targeted delivery, and stimulus-responsive nanoparticle loading address core draggability bottlenecks of terpenoids (e.g., poor aqueous solubility, inadequate tumor targeting, uncontrolled in vivo release), as exemplified by AI-empowered delivery system design.

Terpenoids commonly suffer from poor water solubility and low bioavailability. Nanoscale formulations are a key strategy for improving their pharmacokinetic profiles; however, traditional formulation optimization relies on orthogonal experimental de-signs, which are often inefficient. Artificial intelligence (AI) technologies enable intelligent design of nano formulations by establishing nonlinear relationship models between formulation/process parameters and performance characteristics. Addressing common challenges in delivery systems for poorly soluble drugs—particularly terpenoid compounds—such as inadequate water solubility, low bioavailability, and insufficient stability, relevant research has pursued multi-dimensional approaches: On the one hand, artificial neural network (ANN) models, serving as core AI-enabled tools for formulation optimization, have been successfully applied to process screening and parameter optimization for nanomedicines. For instance, using polymer content, drug content, ultrasonic power, and phase ratio as input parameters, with nanoparticle size as the output parameter, modeling based on 40 experimental datasets has clarified the parameter influence patterns for paclitaxel-loaded PLGA-PMBH nanoparticles [148]. Through 32 sets of experimental data and k-fold cross-validation, a model was constructed to identify the optimal formulation for paclitaxel-etoposide co-loaded mPEG-PLGA nanoparticles. This provides direct empirical support for AI-assisted design of nanodelivery systems for terpenoid compounds [149]. On the other hand, the exploration of fundamental delivery technologies provides data support and conceptual insights for the development of terpenoid formulations. For instance, by encapsulating modified starch around terpenoids such as *α*-pinene, linalool, and limonene as subjects, employing modified starch encapsulation followed by spray drying to prepare formulations, systematically investigating their storage stability. For natural flavonoid compounds (such as apigenin) sharing similar physicochemical limitations with terpenoids, delivery systems constructed using supercritical CO_2_ technology significantly enhanced its solubility, permeability, and stability. This offers complementary insights into AI-enabled, diversified design approaches to delivery systems for poorly soluble natural products [150].

To expand the application scope of terpenoids, combinatorial regimens integrating terpenoids with immunotherapy and targeted therapies are being explored. By modulating the tumor immune microenvironment and synergistically inhibiting tumor cell survival signaling pathways, terpenoids may help overcome the limitations of monotherapeutic chemotherapy in MDR reversal. This approach is intended to potentially improve the durability of anti-tumor efficacy and reduce the incidence of resistance-associated recurrence.

Structure–activity relationship (SAR) studies have facilitated the design and development of more potent MDR reversers, and natural products are frequently combined with other chemotherapeutics to potentially enhance therapeutic efficacy and mitigate drug resistance development. The MDR reversal effects of terpenoids vary by structural subtype, which may support the identification of lead compounds in subsequent research. Future investigations could focus on discovering novel natural product-derived MDR reversers, elucidating their underlying mechanisms of action, optimizing their structures to improve efficacy and reduce toxicity, and conducting additional clinical trials to verify their safety and efficacy profiles. Collectively, natural products exhibit considerable potential for MDR reversal, and continued exploration of terpenoid biology may further advance the research and application of natural products, thereby supporting their broader translation into tumor therapy.

## Figures and Tables

**Table 1:** Three generations of tumor multidrug resistance reversal agents.

Classification of Inhibitors	Compounds	Representative Compound Structures	Disadvantages	Clinical Trial Status
First generation	Verapamil Cyclosporine A Vincristine Reserpine Tamoxifen	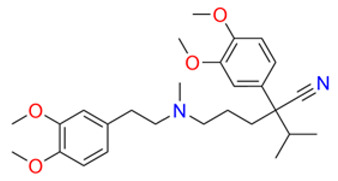	High dosage, poor efficacy and susceptibility to toxic side effects [64]	Verapamil-induced cardiotoxicity and cyclosporine A-induced nephrotoxicity Early Phase I/II trials, self-induced toxicity [65]
Second generation	(R) Verapamil Dexniguldipine Biricodar Trifluoperazine Valspodar(PSC-833)	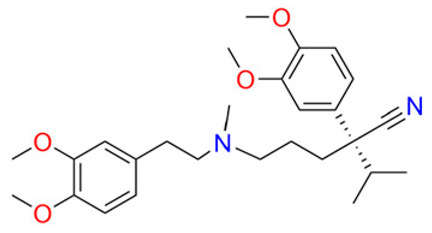	Poor selectivity, disruption of normal cellular metabolism and toxic side effects [66,67]	Interference with CYP3A4 metabolism induces severe pharmacokinetic interactions, and the superimposed toxicities overshadow the therapeutic efficacy PSC 833 Pharmacokinetic Interactions, Phase III Ineffective [68,69]
Third generation	Tariquidar (XR9576) Laniquidar (R101933) Annamycin Zosuquidar (LY335979)	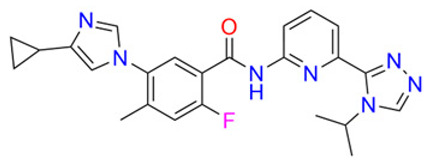	Despite improvements, they remain highly toxic to normal cells and have pharmacokinetic abnormalities [70,71]	The single-target mechanism fails to counteract the multifactorial complexity of clinical multidrug resistance (MDR) Phase III efficacy negative [72]

**Table 2:** Biology of monoterpene compounds.

Number	Source	Configuration	Cell	Mechanism	Reference
1	*Zanthoxyli*	Monoterpenes	LLC-PK1, LLC-GA5 COL150	1. The ethyl acetate extract of Sichuan peppe exhibited the strongest P-gp inhibitory activity, with an IC_50_ of 166 μg/mL; 2. Among monoterpenoid constituents, (R)-(+)-citronellaldehyde and (S)-*β*-citronellol exhibited P-gp IC_50_ values of 167 μM and 504 μM, respectively, significantly enhancing [^3^H] digoxin accumulation within LLC-GA5-COL150 cells.	[92]
2	*Thymus vulgaris*		MCF-7	MTT assays confirmed that apigenin combined with 5-FU exhibited a dose-dependent inhibition of MCF-7 cell proliferation, with IC_50_ values of 135 µM (apigenin) and 102 µM (5-FU), respectively.	[91]
3	*Saffron*	monoterpenoid derivatives	HT-29 HT29/Dx	Combination therapy studies demonstrate that TMPE exhibits synergistic effects with doxorubicin (CI < 1), enhancing LoVo/Dx cell sensitivity to doxorubicin by 40%.	[93]
4	*Cymbopogon citratus*	Monoterpenes	MCF-7/ADR HepG-2/ADR SKOV-3/ADR	Drug accumulation experiments demonstrated that LG (20 µg/mL) enhanced the accumulation of doxorubicin within drug-resistant cells by 2.1 to 2.3-fold and citral by 1.9 to 2.0-fold, exhibiting superior efficacy compared to the positive control verapamil.	[94]
5	*Dryobalanops* *aromatica*			B-DTX-TET-M crosses the blood–brain barrier, accumulates at glioma sites, achieves a tumor suppression rate of 78.3%, and significantly reduces P-gp expression in tumor tissue.	[95]

**Table 3:** Biology of sesquiterpene compounds.

Number	Source	Configuration	Cell	Mechanism	Reference
6, 7	*Leishmania tropica*	Dihydro-*β*-agarofuran-type sesquiterpenoid	Sf 9	Inhibiting the drug efflux pump P-glycoprotein (P-gp)	[98]
14	*Curcuma zedoaria Roscoe*	Sesquiterpene hydrocarbons	MCF-7 DLD-1 L-929	Combination of 5 μM *β*-caryophyllene with paclitaxel reduced the IC_50_ of MCF-7/ADR cells from 4.7 μM to 0.5 μM.	[96]
13	*Ferulaschtschurow skiana*	Sesquiterpene lactones	MDCK-MDR1	10 μM Conferone reduced the IC_50_ of MDCK-MDR1 cells for vincristine from 12.8 μM to 2.3 μM, representing a 5.6-fold reversal.	[99]
8–11	*Illicium simonsii*		MCF-7/ADR Bel7402/5-FU	The reversal ratio for MCF-7/ADR cells reached 12.26, comparable to the activity of verapamil.	[100]
12	*Phellodendron amurense*	Limonoid-type sesquiterpenoids	MES-SA/DX5 HCT15	Obacunone exhibits comparable activity to verapamil in MES-SA/DX5 (ED_50_ = 0.028 μg/mL) and HCT15 (ED_50_ = 0.0011 μg/mL) cells.	[97]

**Table 4:** Biology of diterpenoids compounds.

Number	Source	Configuration	Cell	Mechanism	References
15	*Euphorbia glomerulans*	Jatrophane Ingenane	MCF-7/ADR	The binding affinity to P-gp (−8.4 kcal/mol) is superior to that of VRP (−7.5 kcal/mol).	[101]
16–19	*Euphorbia kansui*		HepG2/ADR	6*β*,7*β*-epoxy-3*β*,4*β*,5*β*-trihydroxy-20-deoxy-gigantol RF = 186.4, 3,5,7,15-tetraacetoxy-9-nicotinoxy-14-oxo-phytol-6 (17),11-diene RF = 143.8, both superior to VRP (RF = 93.7)	[103]
20	*Euphorbia* spp.	Jatrophane Modified jatrophane	MCF-7	At 60 μM, the compound exhibits a FAR of 82.2, outperforming VRP.	[105]
21–22	*Euphorbia mellifera*	Jatrophane	EL4, COLO320 L5178Y/MDR	EpieuphoscopinB IC_50_ = 1.71 μM, superior to VRP (3.37 μM).	[106]
23	*Euphorbia royleana*		HepG2/DOX	Following compound treatment, the nuclear accumulation rate of DOX within MCF-7/ADR cells increased 3.8-fold. RF = 28.7, outperforming VRP (RF = 19.5).	[107]
24–25	*Euphorbia nicaeensis*		NCI-H460/R	Selectivity index (SI) of the compound against P-gp = 28.5. DNA fragmentation rate in drug-resistant cells increased 4.2-fold.	[108]
26	*Jatropha Euphorbiaceae*		K526/R7	The compound exhibits a binding constant K_d = 0.8 μM with P-gp; C-3-position benzyloxy substitution markedly enhances activity.	[109]
27–28	*Euphorbia kansui*	Ingenane	MCF-7/ADR	At 5 μM, glycerol A exhibited an RF of 21.5, while glycerol B at 5 μM yielded an RF of 18.8, both superior to VRP (RF = 6.7).	[110]
29	*Euphorbia*		HepG2/ADR	At 10 μM, the compound exhibits an RF of 35.2, significantly enhancing the toxicity of ADR towards drug-resistant cells.	[111]
30–31	*Euphorbia taurinensis*		L5178 L5178/MDR	Following treatment with the compound, the mitochondrial membrane potential in drug-resistant cells decreased by 45%. RF = 31.6, outperforming VRP (RF = 29.2).	[112]
32	*Euphorbia*	Lathyrane	COLO LoVo/DOX	Latilagascene B at 100 μM exhibits a FIR of 6.51, enhancing the nuclear distribution of DOX.	[113]
33	*Euphorbia*		MCF-7/ADR KBv200	Molecular docking indicates a binding energy of −9.2 kcal/mol with the TMD pocket of ABCB1.	[114]
34–35	*Euphorbiaceae*		HepG2/ADR HCT-15/5-FU	The compounds exhibited RF values of 27.3 and 21.8 for P-gp and BCRP, respectively.	[115]
36–37	*Euphorbia* *portlandica*	Segetane	L5178Y EL4	At 40 μg/mL, compound 1 exhibited a fluorescence activity ratio of R = 40.3, while compound at 40 μg/mL yielded R = 30.7, outperforming VRP (R = 9.7).	[116]
38–41	*Euphorbia*	Ent-abietane	HepG-2/ADR	Compound (helioscopinolide F) at 40 μg/mL yielded R = 138.56, significantly outperforming VRP (R = 12.23).	[104]
42	*Salvia amarissima Lamiaceae*	Triterpenes from Salvia miltiorrhiza (Arabidopsis thaliana AC)	HepG2/ADR	Compound IC_50_ = 3.1 μM, RF = 25.8; Bcl-2 mRNA levels decreased by 71%	[117]
43–47	*Euphorbia*	Taxane diterpenes	MCF-7/ADR KBv200	Following compound treatment, VCR accumulation within MDR cells increased 4.1-fold;	[118]

**Table 5:** Biology of diterpenoids compounds.

Number	Source	Configuration	Cell	Mechanism	References
48	*Euphorbia lathyris*	Lathyrane	HepG2/ADR	The compound significantly reduced the mRNA levels of ABCB1 and ABCC1 in HepG2-ADR cells.	[118]
49–53	*Euphorbia esula*	Jatrophane	HepG2/ADR	Significantly reduced mRNA levels of Nrf2, ABCB1, and ABCG2	[117]
54–58	*Euphorbia jolkinii*		L5178Y	Membrane protein extraction confirmed a 53% and 47% reduction in the membrane localisation levels of P-gp and MRP1, respectively.	[84]

**Table 6:** Biology of diterpenoids compounds.

Number	Source	Configuration	Cell	Experimental Results	References
59	*Euphorbia kansui*	Jatrophane	MCF-7/ADR	IC_50_ = 2.3 μM, RF = 32.1	[85]
60	*Euphorbia macrorrhiza*		KB KBv200	Following treatment with the compound, MDR1 mRNA levels decreased by 62%.	[86]
61	*Euphorbia sororia*		MCF-7/ADR	EC_50_ = 1.82 μM, RF = 26.90	[87]

**Table 7:** Biology of diterpenoids compounds.

Number	Source	Configuration	Cell	Experimental Results	References
62–63	*Euphorbia boetica*	6,17-Epoxy-12-triterpenoid	L5178YT	The epoxy-modified compound exhibited an RF of 41.5, representing a 2.3-fold increase compared to the unmodified form.	[119]
64–65	*Euphorbia glomerulans*	Jatrophane	MCF-7 MCF-7/ADR	Following treatment with the compound, P-gp ATPase activity decreased by 67%.	[120]
66–70	*Euphorbia*	Jatrophane	MDA-MB-231	At a concentration of 10 μM, the compound exhibited reversal rates of 78% and 65% for MDR1 and MRP, respectively.	[121]
71	*Euphorbia boetica*	Jatrophane	L5178Y-MDR Colo320	RF = 36.4, superior to VRP (RF = 29.3)	[102]
72–78	*Euphorbia*	Jatrophane	ML5178	Synergy Index (CI) = 0.42, indicating a significant synergistic effect	[122]

**Table 8:** Biology of diterpenoids compounds.

Number	Source	Type	Cell	Experimental Results	Refereence
79	*Euphorbia pedroi*	spiropedroxodiol	L5178YMDRColo320	At a concentration of 0.2 μM, spiropedroxodiol exhibited a fluorescence activity ratio (FAR) of 43.75 against L5178Y-MDR cells, significantly promoting Rh123 accumulation.	[124]
80	*Sipholane*		KB-C2; KB3-1	At a concentration of 5 μM, KB-C2 cells exhibited a reduction in colchicine resistance from 578-fold to 35.6-fold.	[126]
81–82	*Callyspongia*	sipholenol	KB-C2 KB-3-1	The cis-decalin ring system (rings C/D), connected via a methylene bridge, stably embeds within the ABCB1 binding pocket.	[129]
83	*Euphorbia soongarica*	sooneuphanone	KB KBv200	Compound **83** exhibited a FAR of 5.23 at 10 μM, reducing the IC_50_ of KBv200 cells by over 3200-fold when combined with vinorelbine, demonstrating superior reversal activity compared to verapamil;	[123]
84	Rhizoma Alismatis	Alisol B 23-acetate	HepG2-DR; K562-DR	5 μM ABA reduced the IC_50_ of paclitaxel in HepG2-DR cells from 4.8 M to 0.6 μM, with CI < 0.8 (strong synergy).	[125]
85	23-hydroxybetulinic acid	Lupane skeleton	KB-C2	5 μM BBA reversed the resistance of HepG2/ADM cells to doxorubicin by 53.6-fold.	[130]
86	*Carpobrotus edulis*		L5178Y	Compound **86** increased R123 accumulation in L5178Y-MDR cells by 3- to 8-fold at a concentration of 10 μM, with a FAR value of 2.8–7.5.	[127]
87–88	*Momordica balsamina*	Cucurbitane skeleton	L5178Y-MDRMDR1 L5178Y-PAR	At 20 μM, the fold reversal (FAR) value was 198.9, and its combination with doxorubicin resulted in a combination index (CI) of 0.268, indicating strong synergy.	[131]
89	Momordica *L.* *Cucurbitaceae*		COLO 205 COLO 320	When combined with doxorubicin, the survival rate of KB-C2 cells was reduced from 62% to 18%, with a combination index (CI) ranging from 0.32 to 0.67, indicating synergistic effects. Molecular docking analysis revealed that key hydrogen bonds were formed with Tyr310 and Phe728 residues of ABCB1.	[132]
90–91	*Salvia* *Lamiaceae*	Oleanane skeleton	KB-C2 KB/MRP MRP1	At 20 μM, ursolic acid reduced the IC_50_ of doxorubicin in KBv200 cells from 46 μM to 2.3 μM, representing a 19.9-fold reversal.	[133]
92	*Euphorbia species*		Ma104	At a concentration of 15 μM, quinic acid reduced the IC_50_ of vincristine by 6.8-fold in KB-CV60 cells overexpressing ABCC1.	[134]
93	*Euphorbia species*		LLC-PK1 Sf9	At a concentration of 5 μM, mitoxantrone fluorescence intensity increased by 4–9-fold in cells overexpressing ABCG2.	[135]

## Data Availability

No new data were created or analyzed in this study. Data sharing is not applicable to this article.

## References

[1] Chen W., Zheng R., Baade P.D., Zhang S., Zeng H., Bray F., Jemal A., Yu X.Q., He J. (2016). Cancer statistics in China, 2015. CA Cancer J. Clin..

[2] Bizuayehu H.M., Ahmed K.Y., Kibret G.D., Dadi A.F., Belachew S.A., Bagade T., Tegegne T.K., Venchiarutti R.L., Kibret K.T., Hailegebireal A.H. (2024). Global Disparities of Cancer and Its Projected Burden in 2050. JAMA Netw. Open.

[3] Li Y., Huang Y., Huang H., Wei T., Zhang A., Xing L., Yin X., Li H., Ren G., Li F. (2025). Global, regional, and national burden of male breast cancer in 204 countries and territories: A systematic analysis from the global burden of disease study, 1990–2021. eClinicalMedicine.

[4] Chen Y., Chen Z., Chen R., Fang C., Zhang C., Ji M., Yang X. (2022). Immunotherapy-based combination strategies for treatment of EGFR-TKI-resistant non-small-cell lung cancer. Future Oncol..

[5] Zhang L., Song J., Kong L., Yuan T., Li W., Zhang W., Hou B., Lu Y., Du G. (2020). The strategies and techniques of drug discovery from natural products. Pharmacol. Ther..

[6] Zhang L., Ye B., Chen Z., Chen Z.-S. (2022). Progress in the studies on the molecular mechanisms associated with multidrug resistance in cancers. Acta Pharm. Sin. B.

[7] Kawase M., Motohashi N. (2003). New multidrug resistance reversal agents. Curr. Drug Targets.

[8] Guo B.-J., Bian Z.-X., Qiu H.-C., Wang Y.-T., Wang Y. (2017). Biological and clinical implications of herbal medicine and natural products for the treatment of inflammatory bowel disease. Ann. N. Y. Acad. Sci..

[9] Giddings E.L., Champagne D.P., Wu M.-H., Laffin J.M., Thornton T.M., Valenca-Pereira F., Culp-Hill R., Fortner K.A., Romero N., East J. (2021). Mitochondrial ATP fuels ABC transporter-mediated drug efflux in cancer chemoresistance. Nat. Commun..

[10] Ibrahim M.A.A., Abdeljawaad K.A.A., Jaragh-Alhadad L.A., Oraby H.F., Atia M.A.M., Alzahrani O.R., Mekhemer G.A.H., Moustafa M.F., Shawky A.M., Sidhom P.A. (2023). Potential drug candidates as P-glycoprotein inhibitors to reverse multidrug resistance in cancer: An in silico drug discovery study. J. Biomol. Struct. Dyn..

[11] Chang Y.-T., Lin Y.-C., Sun L., Liao W.-C., Wang C.C.N., Chou C.-Y., Morris-Natschke S.L., Lee K.-H., Hung C.-C. (2020). Wilforine resensitizes multidrug resistant cancer cells via competitive inhibition of P-glycoprotein. Phytomedicine.

[12] Liu M., Xu C., Qin X., Liu W., Li D., Jia H., Gao X., Wu Y., Wu Q., Xu X. (2022). DHW-221, a Dual PI3K/mTOR Inhibitor, Overcomes Multidrug Resistance by Targeting P-Glycoprotein (P-gp/ABCB1) and Akt-Mediated FOXO3a Nuclear Translocation in Non-small Cell Lung Cancer. Front. Oncol..

[13] Mohammad I.S., He W., Yin L. (2018). Understanding of human ATP binding cassette superfamily and novel multidrug resistance modulators to overcome MDR. Biomed. Pharmacother..

[14] Kolata G. (1986). Why do cancer cells resist drugs?. Science.

[15] Mehla J., Ernst R., Moore R., Wakschlag A., Marquis M.K., Ambudkar S.V., Golin J. (2014). Evidence for a molecular diode-based mechanism in a multispecific ATP-binding cassette (ABC) exporter: SER-1368 as a gatekeeping residue in the yeast multidrug transporter Pdr5. J. Biol. Chem..

[16] Pote M.S., Gacche R.N. (2023). ATP-binding cassette efflux transporters and MDR in cancer. Drug Discov. Today.

[17] Gottesman M.M., Fojo T., Bates S.E. (2002). Multidrug resistance in cancer: Role of ATP-dependent transporters. Nat. Rev. Cancer.

[18] Leslie E.M., Deeley R.G., Cole S.P. (2005). Multidrug resistance proteins: Role of P-glycoprotein, MRP1, MRP2, and BCRP (ABCG2) in tissue defense. Toxicol. Appl. Pharmacol..

[19] Longley D.B., Johnston P.G. (2005). Molecular mechanisms of drug resistance. J. Pathol..

[20] Liu C., Zhang T., Chen L., Chen Y. (2020). The choice of anti-tumor strategies based on micromolecules or drug loading function of biomaterials. Cancer Lett..

[21] Ramirez M., Rajaram S., Steininger R.J., Osipchuk D., Roth M.A., Morinishi L.S., Evans L., Ji W., Hsu C.H., Thurley K. (2016). Altschuler, Diverse drug-resistance mechanisms can emerge from drug-tolerant cancer persister cells. Nat. Commun..

[22] Awad M.M., Katayama R., McTigue M., Liu W., Deng Y.-L., Brooun A., Friboulet L., Huang D., Falk M.D., Timofeevski S. (2013). Acquired resistance to crizotinib from a mutation in CD74-ROS1. N. Engl. J. Med..

[23] Montagut C., Dalmases A., Bellosillo B., Crespo M., Pairet S., Iglesias M., Salido M., Gallen M., Marsters S., Tsai S.P. (2012). Albanell, Identification of a mutation in the extracellular domain of the Epidermal Growth Factor Receptor conferring cetuximab resistance in colorectal cancer. Nat. Med..

[24] Johnston P.G., Lenz H.J., Leichman C.G., Danenberg K.D., Allegra C.J., Danenberg P.V., Leichman L. (1995). Thymidylate synthase gene and protein expression correlate and are associated with response to 5-fluorouracil in human colorectal and gastric tumors. Cancer Res..

[25] Longley D.B., Ferguson P.R., Boyer J., Latif T., Lynch M., Maxwell P., Harkin D.P., Johnston P.G. (2001). Characterization of a thymidylate synthase (TS)-inducible cell line: A model system for studying sensitivity to TS- and non-TS-targeted chemotherapies. Clin. Cancer Res..

[26] Chu E., Allegra C.J. (1996). The role of thymidylate synthase in cellular regulation. Adv. Enzym. Regul..

[27] Li L.-Y., Guan Y.-D., Chen X.-S., Yang J.-M., Cheng Y. (2020). DNA Repair Pathways in Cancer Therapy and Resistance. Front. Pharmacol..

[28] Huang R., Zhou P.-K. (2021). DNA damage repair: Historical perspectives, mechanistic pathways and clinical translation for targeted cancer therapy. Signal Transduct. Target. Ther..

[29] Mori R., Yoshida K., Futamura M., Suetsugu T., Shizu K., Tanahashi T., Tanaka Y., Matsuhashi N., Yamaguchi K. (2019). The inhibition of thymidine phosphorylase can reverse acquired 5FU-resistance in gastric cancer cells. Gastric Cancer.

[30] Ahmed S., Alam W., Aschner M., Alsharif K.F., Albrakati A., Saso L., Khan H. (2022). Natural products targeting the ATR-CHK1 signaling pathway in cancer therapy. Biomed. Pharmacother..

[31] Rundle S., Bradbury A., Drew Y., Curtin N.J. (2017). Targeting the ATR-CHK1 Axis in Cancer Therapy. Cancers.

[32] Hegazy M.-E.F., Dawood M., Mahmoud N., Elbadawi M., Sugimoto Y., Klauck S.M., Mohamed N., Efferth T. (2020). 2*α*-Hydroxyalantolactone from Pulicaria undulata: Activity against multidrug-resistant tumor cells and modes of action. Phytomedicine.

[33] Zhang C., Guo Q., Chen L., Wu Z., Yan X.J., Zou C., Zhang Q., Tan J., Fang T., Rao Q. (2023). A ribosomal gene panel predicting a novel synthetic lethality in non-BRCAness tumors. Signal Transduct. Target. Ther..

[34] Li X.Y., Cui X., Xie C.Q., Wu Y., Song T., He J.D., Feng J., Cui Q.R., Bin J.L., Li Q.Y. (2023). Andrographolide causes p53-independent HCC cell death through p62 accumulation and impaired DNA damage repair. Phytomedicine.

[35] Elayapillai S.P., Dogra S., Lausen J., Parker M., Kennedy A., Benbrook D.M., Moxley K.M., Hannafon B.N. (2024). ATR inhibition increases reliance on PARP-mediated DNA repair revealing an improved therapeutic strategy for cervical cancer. Gynecol. Oncol..

[36] Han F., Qi G., Li R., Peng J., Yan S., Yuan C., Kong B., Ma H. (2025). USP28 promotes PARP inhibitor resistance by enhancing SOX9-mediated DNA damage repair in ovarian cancer. Cell Death Dis..

[37] Zhou D., Zhang F., Kikuchi T., Yao M., Otsuki K., Chen G., Li W., Li N. (2022). Lathyrane and Jatrophane Diterpenoids from Euphorbia helioscopia Evaluated for Cytotoxicity against a Paclitaxel-Resistant A549 Human Lung Cancer Cell Line. J. Nat. Prod..

[38] Guan J., Chen J. (2013). Mesenchymal stem cells in the tumor microenvironment. Biomed. Rep..

[39] Jena B.C., Mandal M. (2021). The emerging roles of exosomes in anti-cancer drug resistance and tumor progression: An insight towards tumor-microenvironment interaction. Biochim. Biophys. Acta Rev. Cancer.

[40] Nishimura K., Semba S., Aoyagi K., Sasaki H., Yokozaki H. (2012). Mesenchymal stem cells provide an advantageous tumor microenvironment for the restoration of cancer stem cells. Pathobiology.

[41] Bai R., Cui J. (2022). Development of Immunotherapy Strategies Targeting Tumor Microenvironment Is Fiercely Ongoing. Front. Immunol..

[42] Tufail M., Wan W.-D., Jiang C., Li N. (2024). Targeting PI3K/AKT/mTOR signaling to overcome drug resistance in cancer. Chem.-Biol. Interact..

[43] Wang J., Hu K., Cai X., Yang B., He Q., Wang J., Weng Q. (2022). Targeting PI3K/AKT signaling for treatment of idiopathic pulmonary fibrosis. Acta Pharm. Sin. B.

[44] Zhang W., Li S., Li C., Li T., Huang Y. (2022). Remodeling tumor microenvironment with natural products to overcome drug resistance. Front. Immunol..

[45] Emran T.B., Shahriar A., Mahmud A.R., Rahman T., Abir M.H., Siddiquee M.F., Ahmed H., Rahman N., Nainu F., Wahyudin E. (2022). Multidrug Resistance in Cancer: Understanding Molecular Mechanisms, Immunoprevention and Therapeutic Approaches. Front. Oncol..

[46] Zeng A.-Q., Chen X., Dai Y., Zhao J.-N. (2024). Betulinic acid inhibits non-small cell lung cancer by repolarizing tumor-associated macrophages via mTOR signaling pathway. China J. Chin. Mater. Medica.

[47] Evans L., Milward K., Attanoos R., Clayton A., Errington R., Tabi Z. (2021). Macrophage Plasticity and Function in the Lung Tumour Microenvironment Revealed in 3D Heterotypic Spheroid and Explant Models. Biomedicines.

[48] Chen B., Qiu X., Li Y. (2025). Exosomes in ovarian cancer: Impact on drug resistance and advances in SERS detection techniques. J. Pharm. Anal..

[49] Cheng H.Y., Su G.L., Wu Y.X., Chen G., Yu Z.L. (2024). Extracellular vesicles in anti-tumor drug resistance: Mechanisms and therapeutic prospects. J. Pharm. Anal..

[50] Lim S.M., Syn N.L., Cho B.C., Soo R.A. (2018). Acquired resistance to EGFR targeted therapy in non-small cell lung cancer: Mechanisms and therapeutic strategies. Cancer Treat. Rev..

[51] Li Y., Zhao B., Peng J., Tang H., Wang S., Peng S., Ye F., Wang J., Ouyang K., Li J. (2024). Inhibition of NF-κB signaling unveils novel strategies to overcome drug resistance in cancers. Drug Resist. Updat..

[52] Zhu K., Wu Y., He P., Fan Y., Zhong X., Zheng H., Luo T. (2022). PI3K/AKT/mTOR-Targeted Therapy for Breast Cancer. Cells.

[53] Wu S., Liao X., Zhu Z., Huang R., Chen M., Huang A., Zhang J., Wu Q., Wang J., Ding Y. (2022). Antioxidant and anti-inflammation effects of dietary phytochemicals: The Nrf2/NF-κB signalling pathway and upstream factors of Nrf2. Phytochemistry.

[54] Cao Y., Yi Y., Han C., Shi B. (2024). NF-κB signaling pathway in tumor microenvironment. Front. Immunol..

[55] Su H., Peng C., Liu Y. (2024). Regulation of ferroptosis by PI3K/Akt signaling pathway: A promising therapeutic axis in cancer. Front. Cell Dev. Biol..

[56] Xiong Y., Wen S., Li Y., Wei Y., Fang B., Li C., Huang Q., Lin X. (2022). Comprehensive analysis of transcriptomics and metabolomics to illustrate the underlying mechanism of helenalin against hepatic fibrosis. Eur. J. Pharmacol..

[57] Yang L., Yang M., Zhang H., Wang Z., Yu Y., Xie M., Zhao M., Liu L., Cao L. (2012). S100A8-targeting siRNA enhances arsenic trioxide-induced myeloid leukemia cell death by down-regulating autophagy. Int. J. Mol. Med..

[58] Pan B., Chen D., Huang J., Wang R., Feng B., Song H., Chen L. (2014). HMGB1-mediated autophagy promotes docetaxel resistance in human lung adenocarcinoma. Mol. Cancer.

[59] Fan T., Zhang C., Zong M., Zhao Q., Yang X., Hao C., Zhang H., Yu S., Guo J., Gong R. (2014). Peptidylarginine deiminase IV promotes the development of chemoresistance through inducing autophagy in hepatocellular carcinoma. Cell Biosci..

[60] Sun W.L., Chen J., Wang Y.P., Zheng H. (2011). Autophagy protects breast cancer cells from epirubicin-induced apoptosis and facilitates epirubicin-resistance development. Autophagy.

[61] Sun W.L., Lan D., Gan T.Q., Cai Z.W. (2015). Autophagy facilitates multidrug resistance development through inhibition of apoptosis in breast cancer cells. Neoplasma.

[62] Yang Y., Liu L., Tian Y., Gu M., Wang Y., Ashrafizadeh M., Reza Aref A., Cañadas I., Klionsky D.J., Goel A. (2024). Autophagy-driven regulation of cisplatin response in human cancers: Exploring molecular and cell death dynamics. Cancer Lett..

[63] Fourrier C., Bryksin V., Hattersley K., Hein L.K., Bensalem J., Sargeant T.J. (2021). Comparison of chloroquine-like molecules for lysosomal inhibition and measurement of autophagic flux in the brain. Biochem. Biophys. Res. Commun..

[64] Nobili S., Landini I., Mazzei T., Mini E. (2012). Overcoming tumor multidrug resistance using drugs able to evade P-glycoprotein or to exploit its expression. Med. Res. Rev..

[65] Ozols R.F., Cunnion R.E., Klecker R.W., Hamilton T.C., Ostchega Y., Parrillo J.E., Young R.C. (1987). Verapamil and adriamycin in the treatment of drug-resistant ovarian cancer patients. J. Clin. Oncol..

[66] Duran G.E., Derdau V., Weitz D., Philippe N., Blankenstein J., Atzrodt J., Sémiond D., Gianolio D.A., Macé S., Sikic B.I. (2018). Cabazitaxel is more active than first-generation taxanes in ABCB1(+) cell lines due to its reduced affinity for P-glycoprotein. Cancer Chemother. Pharmacol..

[67] Lhommé C., Joly F., Walker J.L., Lissoni A.A., Nicoletto M.O., Manikhas G.M., Baekelandt M.M., Gordon A.N., Fracasso P.M., Mietlowski W.L. (2008). Phase III study of valspodar (PSC 833) combined with paclitaxel and carboplatin compared with paclitaxel and carboplatin alone in patients with stage IV or suboptimally debulked stage III epithelial ovarian cancer or primary peritoneal cancer. J. Clin. Oncol..

[68] Baer M.R., George S.L., Dodge R.K., O’Loughlin K.L., Minderman H., Caligiuri M.A., Anastasi J., Powell B.L., Kolitz J.E., Schiffer C.A. (2002). Phase 3 study of the multidrug resistance modulator PSC-833 in previously untreated patients 60 years of age and older with acute myeloid leukemia: Cancer and Leukemia Group B Study 9720. Blood.

[69] Boote D.J., Dennis I.F., Twentyman P.R., Osborne R.J., Laburte C., Hensel S., Smyth J.F., Brampton M.H., Bleehen N.M. (1996). Phase I study of etoposide with SDZ PSC 833 as a modulator of multidrug resistance in patients with cancer. J. Clin. Oncol..

[70] Karbanova S., Cerveny L., Jiraskova L., Karahoda R., Ceckova M., Ptackova Z., Staud F. (2019). Transport of ribavirin across the rat and human placental barrier: Roles of nucleoside and ATP-binding cassette drug efflux transporters. Biochem. Pharmacol..

[71] Cui Q., Wang J.Q., Assaraf Y.G., Ren L., Gupta P., Wei L., Ashby C.R., Yang D.H., Chen Z.S. (2018). Modulating ROS to overcome multidrug resistance in cancer. Drug Resist. Updat..

[72] Fox E., Bates S.E. (2007). Tariquidar (XR9576): A P-glycoprotein drug efflux pump inhibitor. Expert Rev. Anticancer. Ther..

[73] Fan J., To K.K.W., Chen Z.-S., Fu L. (2022). ABC transporters affects tumor immune microenvironment to regulate cancer immunotherapy and multidrug resistance. Drug Resist. Updates.

[74] Zhao X., Di J., Luo D., Vaishnav Y., Kamal n., Nuralieva N., Verma D., Verma P., Verma S. (2023). Recent developments of P-glycoprotein inhibitors and its structure–activity relationship (SAR) studies. Bioorg. Chem..

[75] Mollazadeh S., Sahebkar A., Hadizadeh F., Behravan J., Arabzadeh S. (2018). Structural and functional aspects of P-glycoprotein and its inhibitors. Life Sci..

[76] Lima Á.A.N., Koester L.S., Veiga-Junior V.F. (2021). Formulating Bioactive Terpenes. Biomolecules.

[77] Wang J., VanderWeide J., Yan Y., Tindjau R., Pico J., Deluc L., Zandberg W.F., Castellarin S.D. (2022). Impact of hormone applications on ripening-related metabolites in Gewürztraminer grapes (*Vitis vinifera* L.): The key role of jasmonates in terpene modulation. Food Chem..

[78] Zongo A.W.-S., Jin C., Hao G., Yu N., Zogona D., Nie X., Lu Y., Ye Q., Meng X. (2024). Functional compounds of Torreya grandis nuts and their processing byproducts: Extraction process, health benefits, and food applications—A comprehensive review. Food Res. Int..

[79] Kim Y.-J., Choi H.-J., Chung M.-S., Ko M.-J. (2022). Selective extraction of oxygenated terpene in caraway (Carum carvi L.) using subcritical water extraction (SWE) technique. Food Chem..

[80] Mironeasa S., Coţovanu I., Mironeasa C., Ungureanu-Iuga M. (2023). A Review of the Changes Produced by Extrusion Cooking on the Bioactive Compounds from Vegetal Sources. Antioxidants.

[81] Yan H., Li W.-X., Zhu Y.-L., Lin Z.-Y., Chen D., Zhang Y., Lv H.-P., Dai W.-D., Ni D.-J., Lin Z. (2024). Comprehensive comparison of aroma profiles and chiral free and glycosidically bound volatiles in Fujian and Yunnan white teas. Food Chem..

[82] Hanssens J., Meneses D., Saya J.M. (2025). Terpenes and Terpenoids: How can we use them?. Eur. J. Org. Chem..

[83] Ji W., Ji X. (2021). Comparative Analysis of Volatile Terpenes and Terpenoids in the Leaves of Pinus Species—A Potentially Abundant Renewable Resource. Molecules.

[84] Reis M., Ferreira R.J., Santos M.M.M., dos Santos J., Molnár D.J.V.A., Ferreira M.-J.U. (2013). Enhancing Macrocyclic Diterpenes as Multidrug-Resistance Reversers: Structure–Activity Studies on Jolkinol D Derivatives. J. Med. Chem..

[85] Zheng Q., Chen N.-Y., Lou S.-Q., Liu Q.-Q., Luo Y.-T., Liang D.-E., Zhan Z.-J., Ma L.-F. (2022). New Jatrophane-Type Diterpenoids from Euphorbia kansui as Potential MDR Reversal Agents. Chem. Biodivers..

[86] Gao J., Chen Q.-B., Liu Y.-Q., Xin X.-L., Yili A., Aisa H.A. (2016). Diterpenoid constituents of Euphorbia macrorrhiza. Phytochemistry.

[87] Yang H., Mamatjan A., Tang D., Aisa H.A. (2021). Jatrophane diterpenoids as multidrug resistance modulators from *Euphorbia sororia*. Bioorg. Chem..

[88] Liu Q., Cai P., Guo S., Shi J., Sun H. (2020). Identification of a lathyrane-type diterpenoid EM-E-11-4 as a novel paclitaxel resistance reversing agent with multiple mechanisms of action. Aging.

[89] Bautista E., Fragoso-Serrano M., Ortiz-Pastrana N., Toscano R.A., Ortega A. (2016). Structural elucidation and evaluation of multidrug-resistance modulatory capability of amarissinins A-C, diterpenes derived from *Salvia amarissima*. Fitoterapia.

[90] Bergman M.E., Franks A.E., Phillips M.A. (2022). Biosynthesis, natural distribution, and biological activities of acyclic monoterpenes and their derivatives. Phytochem. Rev..

[91] Ghorbanzadeh V., Aljaf K.A.H., Wasman H.M., Pirzeh L., Azimi S., Dariushnejad H. (2022). Carvacrol Enhance Apoptotic Effect of 5-FU on MCF-7 Cell Line via inhibiting P-glycoprotein: An In-silco and In-vitro Study. Drug. Res..

[92] Yoshida N., Takagi A., Kitazawa H., Kawakami J., Adachi I. (2005). Inhibition of P-glycoprotein-mediated transport by extracts of and monoterpenoids contained in Zanthoxyli fructus. Toxicol. Appl. Pharmacol..

[93] Środa-Pomianek K., Palko-Łabuz A., Poła A., Ferens-Sieczkowska M., Wesołowska O., Kozioł A. (2020). TMPE Derived from Saffron Natural Monoterpene as Cytotoxic and Multidrug Resistance Reversing Agent in Colon Cancer Cells. Int. J. Mol. Sci..

[94] Mukhtar M.H., El-Readi M.Z., Elzubier M.E., Fatani S.H., Refaat B., Shaheen U., Adam Khidir E.B., Taha H.H., Eid S.Y. (2023). Cymbopogon citratus and Citral Overcome Doxorubicin Resistance in Cancer Cells via Modulating the Drug’s Metabolism, Toxicity, and Multidrug Transporters. Molecules.

[95] Liu Y., Cai J.Y., Liu Y., Zhang L., Guo R.B., Li X.T., Ma L.Y., Kong L. (2024). Borneol-modified docetaxel plus tetrandrine micelles for treatment of drug-resistant brain glioma. Drug Dev. Ind. Pharm..

[96] Barthomeuf C., Demeule M., Grassi J., Saidkhodjaev A., Beliveau R. (2006). Conferone from Ferula schtschurowskiana enhances vinblastine cytotoxicity in MDCK-MDR1 cells by competitively inhibiting P-glycoprotein transport. Planta Medica.

[97] Min Y.D., Kwon H.C., Yang M.C., Lee K.H., Choi S.U., Lee K.R. (2007). Isolation of limonoids and alkaloids fromPhellodendron amurense and their multidrug resistance (MDR) reversal activity. Arch. Pharmacal Res..

[98] Cortés-Selva F., Jiménez I.A., Munoz-Martínez F., Campillo M., Bazzocchi I.L., Pardo L., Ravelo A.G., Castanys S., Gamarro F. (2005). Dihydro-beta-agarofuran sesquiterpenes: A new class of reversal agents of the multidrug resistance phenotype mediated by P-glycoprotein in the protozoan parasite Leishmania. Curr. Pharm. Des..

[99] Legault J., Pichette A. (2007). Potentiating effect of beta-caryophyllene on anticancer activity of alpha-humulene, isocaryophyllene and paclitaxel. J. Pharm. Pharmacol..

[100] Wei D.-D., Wang J.-S., Kong L.-Y. (2011). Reversal Effects of Components from the Fruits of Illicium simonsii on Human Adriamycin-resistant MCF-7 and 5-Fluorouracil-resistant Bel7402 Cells. Phytother. Res..

[101] Hasan A., Tang D., Nijat D., Yang H.Q., Aisa H.A. (2021). Diterpenoids from Euphorbia glomerulans with potential reversal activities against P-glycoprotein-mediated multidrug resistance. Bioorg. Chem..

[102] Neto S., Duarte N., Pedro C., Spengler G., Molnár J., Ferreira M.-J.U. (2019). Effective MDR reversers through phytochemical study of Euphorbia boetica. Phytochem. Anal..

[103] Wang S.Y., Li J.C., Liu D., Yang T., Chen X.Q., Li R.T. (2021). Ingenane and jatrophane-type diterpenoids from *Euphorbia kansui* with multidrug resistance reversal activity. Phytochemistry.

[104] Ferreira M.J., Gyémánt N., Madureira A.M., Molnár J. (2005). Inhibition of P-glycoprotein transport activity in a resistant mouse lymphoma cell line by diterpenic lactones. Anticancer Res..

[105] Corea G., Di Pietro A., Dumontet C., Fattorusso E., Lanzotti V. (2009). Jatrophane diterpenes from Euphorbia spp. as modulators of multidrug resistance in cancer therapy. Phytochem. Rev..

[106] Valente I., Reis M., Duarte N., Serly J., Molnár J., Ferreira M.-J.U. (2012). Jatrophane Diterpenes from Euphorbia mellifera and Their Activity as P-Glycoprotein Modulators on Multidrug-Resistant Mouse Lymphoma and Human Colon Adenocarcinoma Cells. J. Nat. Prod..

[107] Shaker S., Sang J., Yan X.L., Fan R.Z., Tang G.H., Xu Y.K., Yin S. (2020). Diterpenoids from Euphorbia royleana reverse P-glycoprotein-mediated multidrug resistance in cancer cells. Phytochemistry.

[108] Krstić G., Jadranin M., Todorović N.M., Pešić M., Stanković T., Aljančić I.S., Tešević V.V. (2018). Jatrophane diterpenoids with multidrug-resistance modulating activity from the latex of Euphorbia nicaeensis. Phytochemistry.

[109] Corea G., Fattorusso E., Lanzotti V., Taglialatela-Scafati O., Appendino G., Ballero M., Simon P.-N., Dumontet C., Di Pietro A. (2003). Jatrophane diterpenes as P-glycoprotein inhibitors. First insights of structure-activity relationships and discovery of a new, powerful lead. J. Med. Chem..

[110] Chen Y., Luo D., Chen N.Y., Zhang Y., Liang D.E., Zhan Z.J., Ma L.F. (2021). New ingenane diterpenoids from Euphorbia kansui reverse multi-drug resistance. Phytochem. Lett..

[111] Zhang Y., Fan R.Z., Sang J., Tian Y.J., Chen J.Q., Tang G.H., Yin S. (2020). Ingol diterpenoids as P-glycoprotein-dependent multidrug resistance (MDR) reversal agents from *Euphorbia marginata*. Bioorg. Chem..

[112] Rédei D., Kúsz N., Sátori G., Kincses A., Spengler G., Burián K., Barina Z., Hohmann J. (2018). Bioactive Segetane, Ingenane, and Jatrophane Diterpenes from *Euphorbia taurinensis*. Planta Medica.

[113] Wiśniewski J., Wesołowska O., Środa-Pomianek K., Paprocka M., Bielawska-Pohl A., Krawczenko A., Duarte N., Ferreira M.-J.U., Duś D., Michalak K. (2016). Euphorbia Species-derived Diterpenes and Coumarins as Multidrug Resistance Modulators in Human Colon Carcinoma Cells. Anticancer Res..

[114] Zhang J.-Y., Mi Y.-J., Chen S.-P., Wang F., Liang Y.-J., Zheng L.-S., Shi C.-J., Tao L.-Y., Chen L.-M., Chen H.-B. (2011). Euphorbia factor L1 reverses ABCB1-mediated multidrug resistance involving interaction with ABCB1 independent of ABCB1 downregualtion. J. Cell. Biochem..

[115] Li W., Tang Y.-Q., Sang J., Fan R.-Z., Tang G.-H., Yin S. (2019). Jatrofolianes A and B: Two Highly Modified Lathyrane Diterpenoids from Jatropha gossypiifolia. Org. Lett..

[116] Madureira A.M., Gyémant N., Ascenso J.R., Abreu P.M., Molnar J., Ferreira M.J. (2006). Euphoportlandols A and B, tetracylic diterpene polyesters from Euphorbia portlandica and their anti-MDR effects in cancer cells. J. Nat. Prod..

[117] Xie X.-L., Fan R.-Z., Hu R., Luo S.-Y., Tang G.-H., Yin S. (2020). Euphoresulanes A-M, structurally diverse jatrophane diterpenoids from *Euphorbia esula*. Bioorg. Chem..

[118] Yang T., Wang S., Li H., Zhao Q., Yan S., Dong M., Liu D., Chen X., Li R. (2020). Lathyrane diterpenes from Euphorbia lathyris and the potential mechanism to reverse the multi-drug resistance in HepG2/ADR cells. Biomed. Pharmacother..

[119] Vieira C., Duarte N., Reis M.A., Spengler G., Madureira A.M., Molnár J., Ferreira M.J. (2014). Improving the MDR reversal activity of 6,17-epoxylathyrane diterpenes. Bioorg. Med. Chem..

[120] Ferreira M.-J.U., Gyémánt N., Madureira A.M., Tanaka M., Koós K., Didziapetris R., Molnár J. (2005). The effects of jatrophane derivatives on the reversion of MDR1- and MRP-mediated multidrug resistance in the MDA-MB-231 (HTB-26) cell line. Anticancer Res..

[121] Hasan A., Liu G.-Y., Hu R., Aisa H.A. (2019). Jatrophane Diterpenoids from Euphorbia glomerulans. J. Nat. Prod..

[122] Duarte N., Járdánházy A., Molnár J., Hilgeroth A., Ferreira M.-J.U. (2008). Synergistic interaction between p-glycoprotein modulators and epirubicine on resistant cancer cells. Bioorg. Med. Chem..

[123] Gao J., Aisa H.A. (2017). Terpenoids from Euphorbia soongarica and Their Multidrug Resistance Reversal Activity. J. Nat. Prod..

[124] Ferreira R.J., Kincses A., Gajdács M., Spengler G., dos Santos D.J.V.A., Molnár J., Ferreira M.-J.U. (2018). Terpenoids from Euphorbia pedroi as Multidrug-Resistance Reversers. J. Nat. Prod..

[125] Wang C., Zhang J.-X., Shen X.-L., Wan C.-K., Tse A.K.-W., Fong W.-F. (2004). Reversal of P-glycoprotein-mediated multidrug resistance by Alisol B 23-acetate. Biochem. Pharmacol..

[126] Jain S., Laphookhieo S., Shi Z., Fu L.-w., Akiyama S.-i., Chen Z.-S., Youssef D.T.A., van Soest R.W.M., El Sayed K.A. (2007). Reversal of P-Glycoprotein-Mediated Multidrug Resistance by Sipholane Triterpenoids. J. Nat. Prod..

[127] Martins A., Vasas A., Schelz Z., Viveiros M., Molnár J., Hohmann J., Amaral L. (2010). Constituents of Carpobrotus edulis inhibit P-glycoprotein of MDR1-transfected mouse lymphoma cells. Anticancer Res..

[128] Huang X., Li G., Li H., Zhong W., Jiang G., Cai J., Xiong Q., Wu C., Su K., Huang R. (2024). Glycyrrhetinic Acid as a Hepatocyte Targeting Ligand-Functionalized Platinum(IV) Complexes for Hepatocellular Carcinoma Therapy and Overcoming Multidrug Resistance. J. Med. Chem..

[129] Jain S., Abraham I., Carvalho P., Kuang Y.-H., Shaala L.A., Youssef D.T.A., Avery M.A., Chen Z.-S., El Sayed K.A. (2009). Sipholane Triterpenoids: Chemistry, Reversal of ABCB1/P-Glycoprotein-Mediated Multidrug Resistance, and Pharmacophore Modeling. J. Nat. Prod..

[130] Zhang D.-M., Shu C., Chen J.-J., Sodani K., Wang J., Bhatnagar J., Lan P., Ruan Z.-X., Xiao Z.-J., Ambudkar S.V. (2012). BBA, a Derivative of 23-Hydroxybetulinic Acid, Potently Reverses ABCB1-Mediated Drug Resistance In Vitro and In Vivo. Mol. Pharm..

[131] Ramalhete C., Mulhovo S., Molnar J., Ferreira M.-J.U. (2016). Triterpenoids from Momordica balsamina: Reversal of ABCB1-mediated multidrug resistance. Bioorg. Med. Chem..

[132] Silva C.D., Ramalhete C., Spengler G., Mulhovo S., Molnar J., Ferreira M.-J.U. (2022). Triterpenes from Momordica balsamina (*African pumpkin*): ABCB1 inhibition and synergistic interaction with doxorubicin in resistant cancer cells. Phytochemistry.

[133] Nabekura T., Yamaki T., Hiroi T., Ueno K., Kitagawa S. (2009). Inhibition of anticancer drug efflux transporter P-glycoprotein by rosemary phytochemicals. Pharmacol. Res..

[134] Braga F., Ayres-Saraiva D., Gattass C.R., Capella M.A.M. (2006). Oleanolic acid inhibits the activity of the multidrug resistance protein ABCC1 (MRP1) but not of the ABCB1 (P-glycoprotein): Possible use in cancer chemotherapy. Cancer Lett..

[135] Yoshida N., Takada T., Yamamura Y., Adachi I., Suzuki H., Kawakami J. (2008). Inhibitory effects of terpenoids on multidrug resistance-associated protein 2- and breast cancer resistance protein-mediated transport. Drug Metab. Dispos..

[136] Srovnalova A., Gurska S., Urban M., Sarek J., Rehulka J., Dzubak P., Hajduch M. (2022). Abstract 1530: Derivatives of betulinic acid act as modulators of the androgen receptor and report cytotoxicity towards cancer cell lines. Cancer Res..

[137] Hordyjewska A., Ostapiuk A., Horecka A., Kurzepa J. (2019). Betulin and betulinic acid: Triterpenoids derivatives with a powerful biological potential. Phytochem. Rev..

[138] Benary G.E., Frank K., Sascha K., Jürgen S. (2025). Monophthalates of betulinic acid and related pentacyclic triterpenes inhibit efficiently the SOS-mediated nucleotide exchange and impact PI3K/AKT signaling in oncogenic K-RAS4B proteins. RSC Adv..

[139] Zhao M., Yang Y., Nian Q., Shen C., Xiao X., Liao W., Zheng Q., Zhang G., Chen N., Gong D. (2023). Phytochemicals and mitochondria: Therapeutic allies against gastric cancer. Phytomedicine.

[140] Zhong H., Han L., Lu R.-Y., Wang Y. (2022). Antifungal and Immunomodulatory Ingredients from Traditional Chinese Medicine. Antibiotics.

[141] Huang D., Zheng D., Sun C., Fu M., Wu Y., Wang H., Yu J., Yang Y., Li Y., Wan X. (2024). Combined multi-omics approach to analyze the flavor characteristics and formation mechanism of gabaron green tea. Food Chem..

[142] Dodoš T., Novaković J., Vujisić L., Marin P.D., Rajčević N. (2024). Geographic variability of winter savory essential oil. Ind. Crops Prod..

[143] Budiman A., Ivana H., Huang K.A., Huang S.A., Nadhira M.S., Rusdin A., Aulifa D.L. (2025). Biocompatible Natural Polymer-Based Amorphous Solid Dispersion System Improving Drug Physicochemical Properties, Stability, and Efficacy. Polymers.

[144] Jin X., Qian H., Xie Y., Liu C., Cheng Y., Hou J., Zheng J. (2025). Biocompatible and Biodegradable Nanocarriers for Targeted Drug Delivery in Precision Medicine. Biomimetics.

[145] Wargantiwar S., Bhattacharya S. (2024). Biocompatible Natural Polymers and Cutting-Edge Fabrication Techniques in the Development of Next-Generation Oral Thin Films for Enhanced Drug Delivery Systems. Curr. Pharm. Des..

[146] Biris M., Gluhovschi A., Boruga M., Lazureanu V., Moza I., Boruga O., Vlaicu B., Poenaru M. (2019). Encapsulation of Three Different Crude Extracts Rich in Triterpenes in a Biocompatible Polymer and their Evaluation in vitro for further Applications as Anti-inflammatory and/or Anti-tumour Alternative Therapeutic Agents. Mater. Plast..

[147] Malík M., Velechovský J., Tlustoš P. (2021). Natural pentacyclic triterpenoid acids potentially useful as biocompatible nanocarriers. Fitoterapia.

[148] Mostafavi S.H., Aghajani M., Amani A., Darvishi B., Noori Koopaei M., Pashazadeh A.M., Maghazei M.S., Alvandifar F., Nabipour I., Karami F. (2014). Optimization of paclitaxel-loaded poly (d,l-lactide-co-glycolide-N-p-maleimido benzoic hydrazide) nanoparticles size using artificial neural networks. Pharm. Dev. Technol..

[149] Maleki H., Naghibzadeh M., Amani A., Adabi M., Khosravani M. (2019). Preparation of Paclitaxel and Etoposide Co-loaded mPEG-PLGA Nanoparticles: An Investigation with Artificial Neural Network. J. Pharm. Innov..

[150] Stasiłowicz-Krzemień A., Rosiak N., Racaniello G.F., Denora N., Cielecka-Piontek J. (2025). Effective and Stable Senomorphic Apigenin Delivery System Obtained by Supercritical Carbon Dioxide Processing. Int. J. Mol. Sci..

